# Defining solute carrier transporter signatures of murine immune cell subsets

**DOI:** 10.3389/fimmu.2023.1276196

**Published:** 2023-11-24

**Authors:** Tania Løve Aaes, Javier Burgoa Cardás, Kodi S. Ravichandran

**Affiliations:** ^1^ Department of Biomedical Molecular Biology, Cancer Research Institute Ghent (CRIG), Ghent University, Ghent, Belgium; ^2^ Unit for Cell Clearance in Health and Disease, VIB-UGent Center for Inflammation Research, Ghent, Belgium; ^3^ Department of Microbiology, Immunology, and Cancer Biology, University of Virginia, Charlottesville, VA, United States; ^4^ Division of Immunobiology, Department of Pathology and Immunology, Washington University School of Medicine, St. Louis, MO, United States

**Keywords:** solute carriers (SLCs), membrane transporter, transcriptome (RNA-seq), phagocytes, innate immunity, Triwise (R), inflammation

## Abstract

Solute carrier (SLC) transporters are membrane-bound proteins that facilitate nutrient transport, and the movement across cellular membranes of various substrates ranging from ions to amino acids, metabolites and drugs. Recently, SLCs have gained increased attention due to their functional linkage to innate immunological processes such as the clearance of dead cells and anti-microbial defense. Further, the druggable nature of these transporters provides unique opportunities for improving outcomes in different immunological diseases. Although the SLCs represent the largest group of transporters and are often identified as significant hits in omics data sets, their role in immunology has been insufficiently explored. This is partly due to the absence of tools that allow identification of SLC expression in particular immune cell types and enable their comparison before embarking on functional studies. In this study, we used publicly available RNA-Seq data sets to analyze the transcriptome in adaptive and innate immune cells, focusing on differentially and highly expressed SLCs. This revealed several new insights: first, we identify differentially expressed SLC transcripts in phagocytes (macrophages, dendritic cells, and neutrophils) compared to adaptive immune cells; second, we identify new potential immune cell markers based on SLC expression; and third, we provide user-friendly online tools for researchers to explore SLC genes of interest (and the rest of the genes as well), in three-way comparative dot plots among immune cells. We expect this work to facilitate SLC research and comparative transcriptomic studies across different immune cells.

## 1 Introduction

Immune cells are bathed in small molecules such as metabolites, hormones, and peptides that circulate either through the blood or the lymph, and encounter many similar molecules within the extracellular spaces of lymphoid and non-lymphoid tissues. Further, these metabolites provide key biomass that can be taken up and used as nutrients as well as for intra- or intercellular communication. Since many of these metabolites and small molecules are charged and cannot freely cross the plasma membrane, cells use specific transporters for the transfer of small molecules across biological membranes.

Transporters of the solute carrier (SLC) superfamily mediate solute influx and efflux across the plasma membrane and intracellular membranes. Currently, >400 SLCs are identified in the human genome (1), making it the second-largest membrane bound protein family after the G protein coupled receptor (GPCR) family (~1,400 members). During homeostasis, there is a constant turnover of metabolites such as sugars, fatty acids, and amino acids within cells, all of which need to be transported into and out of organelles and cells. SLC transporters promote cross-membrane movement of charged organic and inorganic solutes in immune cells.

More than 100 SLC genes are linked to human genetic disorders (2), and the functions of SLCs are linked to many biological processes. However, despite the genetic and functional richness, SLCs are remarkably understudied (3). Further, metabolic changes in immune cells (so-called ‘immunometabolism’) are not only crucial during homeostasis, but also for the differentiation and function of immune cells during inflammation (4–7). Thus, a better appreciation of the SLC family within immune cell populations would help define how immune cells use certain metabolites and small molecules for coordinating different aspects of an immune response, understanding metabolite-based communication between T cells, macrophages, and dendritic cells, and to manipulate particular SLCs to modify specific immune responses.

Here, using publicly available gene expression data sets (RNA-Seq), we first derive new insights into the expression patterns of SLCs in the phagocytic myeloid populations versus B and T lymphocytes. Second, we focus more directly on the SLCs of three major phagocytic cell types: macrophages, dendritic cells, and neutrophils. Using a bioinformatic tool, Triwise, which allows three-way comparisons between transcriptomes, we have designed online interactive plots, through which researchers can explore the expression of a gene of interest and easily assess its relative expression and statistical significance between three subsets of immune cells. Our investigations reveal that SLCs are expressed significantly higher in professional phagocytes compared to B and T lymphocytes, and allude to a significant role for SLC11A1 (also known as NRAMP1) in phagocytes. Specific to macrophage subsets deriving from various tissues, the heme transporter SLC48A1 (also known as HRG1) was highly upregulated compared to other professional phagocytes or adaptive immune cells. Finally, it may be possible to use the high transcript levels of specific SLCs as novel markers for specific dendritic cell subsets, and we could confirm a role for amino acid transporters (SLC3/SLC7 family) in neutrophils in inflammatory settings.

## 2 Materials and methods

### 2.1 Bulk RNA-seq data sets

Open access RNA-seq data sets were retrieved from the Gene Expression Omnibus database (8) or through the ImmGen Data browser (9). The selected data sets, namely, GSE109125 (10), GSE122108 (11, 12), GSE164255 (13) and GSE107011 (14) had the following characteristics: GSE109125 contained murine transcriptome data from cells of the adaptive (e.g. lymphocytes) and innate immune system (e.g. mononuclear phagocytes, innate lymphoid cells and granulocytes), while GSE122108 contained murine data specifically from mononuclear phagocytes. Samples included several tissues allowing for cross-tissue comparisons. Samples were retrieved from untreated mice except for one set of peritoneal neutrophils under an inflammation induced treatment (i.e., Thioglycolate 3%) in GSE109125. The GSE164255 data set included *Salmonella* infectious model samples from spleens of mice and consisted of naïve classical monocytes and both bystander and infected iNOS macrophages, 24 hours post-infection. The GSE107011 data set contained human T and B lymphocytes, neutrophils, monocytes and dendritic cells. The data sets were downloaded in the raw gene-count table format. All sequencing and mapping procedures were performed by the respective authors of the data sets. Briefly, these procedures consisted in: RNA-seq using the standard ImmGen ultra-low-input protocol (GSE122108), the low-input protocol (GSE109125), TRIzol^®^ isolation protocol (GSE107011) or PCR purification beads followed by mRNA processing (GSE164255) (15). All murine samples were sequenced using the Illumina NextSeq 500 and human samples through Illumina HiSeq 2000. GSE109125 samples followed a thorough trimming procedure using sickle (v 1.2) and TrimGalore (v 0.4.0). Reads were mapped to the mm10 (GSE109125 and GSE109125) or mm9 (GSE164255) mouse genome or transcriptome (GSE107011) using hisat2 (16) (GSE109125), STAR (17) (GSE122108) or kallisto (18) (GSE107011). GSE109125 low quality reads (MAPQ < 5) were removed using samtools and duplicated reads were removed using Picard MarkDuplicates function (19).

### 2.2 Microarray data set

The microarray data set GSE35449 (20) containing macrophages in classical and alternative polarization conditions was downloaded from GEO as a normalized count matrix. Please see Beyer et al., PLOS One 2012 (20) for details of M1 and M2 polarization and related RNA analysis.

### 2.3 Analysis of RNAseq data sets

After retrieving the raw gene-count tables, preprocessing and analysis were performed equally to all data sets to ensure consistency. All analyses were performed independently for each data set to avoid batch correction. R4.2.3 (R Foundation for Statistical Computing, Vienna, Austria) with DESeq2 (v 1.38.3) (21) and edgeR (v 3.40.2) (22) packages were used throughout the processing and statistical analyses. An initial filtering step removed all samples that were of no use for this manuscript, leaving 48 total replicates belonging to 23 samples in GSE109125 and 46 replicates from 13 samples in GSE122108. We ensured having at least two replicates per sample, with one exception (**Table 1**). Genes with low read counts across samples were removed with the edgeR filterByExp function using a minimum number of CPM counts of 5 (GSE109125, GSE122108 and GSE107011) or 10 (GSE164255). This led to a reasonable number of genes per data set: 12,077 genes for GSE122108, 18,896 for GSE109125, 9,985 for GSE164255 and 10,403 in GSE107011.

**Table 1:** Data sets used and cellular samples analyzed in this manuscript.

MURINE TRANSCRIPTS							
GEO		Cell type	Tissue	Subtype	Sample name	Cell sorting markers	n
**GSE122108**	ImmGen ULI: OpenSource Mononuclear Phagocytes Project	Macrophages	Thymus	Macrophage	MF.64p.Th	Lin^-^ F4/80^+^ CD64^+^	4
			Spleen	Macrophage	MF.480p.SP	CD19^-^ Ly6G^-^ CD115^-^ CD45^+^ CD11b^lo^ F4/80^+^ MerTK^+^ CD64^+^	2
			Liver	Kupffer cell	MF.KC.Clec4FpTim4p64p.Lv	Clec4F^+^ Tim4^+^ CD45^+^ F4/80^+^ CD64+	3
			Lung, alveolar	Macrophage	MF.alv.11cp64pSiglecFp.Lu	CD45^+^ CD11b^lo^ CD64+ CD11c^+^ SiglecF^+^	3
			Kidney	Macrophage	MF.6Gn480hi.Kd	CD45^+^ CD11b^+^ MHCII^+^ F4/80^hi^ Lin^-^ Ly6G^-^	2
			Peritoneal cavity	Macrophage	MF.PC.1-6	ICAM2^+^ F4/80^+^	6
		Dendritic cells	Liver	cDC1	DC.cDC1.XCR1p.Lv	CD45^+^ XCR1^+^ CD11c^+^ MHCII^+^	3
			Liver	cDC2	DC.cDC2.172ap.Lv	CD45^+^ CD172a^+^ CD11c^+^ MHCII^+^	4
			Liver	pDC	DC.pDC.120g8p11cintp6Cp.Lv	120g8^+^ CD11c^int^ Ly6C^+^ MHCII^+^	4
**GSE109125**	ImmGen ULI: Systemwide RNA-seq profiles (#1)	Macrophages	Spleen	Macrophage	MF.RP.Sp	Mertk^+^ CD64^+^ CD11b^lo^ F4/80^+^	2
			Lung, alveolar	Macrophage	MF.Alv.Lu	CD45^+^ CD11c^+^ SiglecF^+^	2
			Peritoneal cavity	Macrophage	MF.PC	CD115^+^ CD11b^+^ F4/80^+^ CD102^+^ MHCII^lo^ CD226^+^	4
		Dendritic cells	Spleen	cDC1	DC.8+.Sp	CD45^+^ MHCII^+^ CD11c+ CD8^+^ CD4^-^	3
			Spleen	cDC2	DC.4+.Sp	CD45^+^ MHCII^+^ CD11c^+^ CD8- CD4^+^	3
			Spleen	pDC	DC.pDC.Sp	CD45^lo^ CD11b^+^	2
		Granulocytes	Spleen	Neutrophil	GN.Sp	CD11b^+^ Ly6G^+^	2
			Peritoneal cavity	Neutrophil	GN.Thio.PC	CD11b^+^ Ly6G^+^	2
			Bone marrow	Neutrophil	GN.BM	CD11b^+^ Ly6G^+^	2
			Spleen	Basophil	Ba.Sp	CD11b^+^ CD49b^+^ FcER1a^+^ CD11c^-^ CD4^-^ CD8^-^ CD19^-^ CD117^-^	3
			Spleen	Eosinophil	Eo.Sp	CD11b^+^ SiglecF^+^ FSC^lo^ SSC^hi^ CD11c^-^ CD4^-^ CD8^-^ CD19^-^	2
		Innate lymphoid cells	Spleen	ILC1-like NK cell	NK.27 + 11b-.Sp	CD3^-^ CD19^-^ NK1.1^+^ CD127^-^ CD51^-^ CD49a- DX5^+^ CD11b^-^ CD27^+^	2
			Small intestine	ILC2	ILC2.SI	CD45^+^ CD3^-^ CD19^-^ CD127^+^ KLRG1^+^ ST2^+^	2
			Small intestine, lamina propia	ILC3	ILC3.CCR6+.SI	CD45^lo^ CD3^-^ CD19^-^ NK1.1^-^ Thy1^+^ NKp46^-^ CCR6^+^	2
			Small intestine, lamina propria	ILC3	ILC3.NKp46+.SI	CD45^lo^ CD3^-^ CD19^-^ NK1.1^-^ Thy1^+^ NKp46^+^ CCR6^-^	2
		Lymphocytes	Spleen	B cell	B.Sp	CD19^+^ Igm^+^ TCRb^-^	1
			Spleen	CD4^+^ T cell	T.4.Nve.Sp	CD4^+^ CD8^-^ TCRb^hi^ CD62L^hi^ CD44^lo^ CD25^-^ Dump^-^	2
**GSE164255**	Hoffman et al., Immunity 2021	Monocytes	Spleen	Monocyte	CM	Lin^-^ CD11b^+^ F4/80^-^ MHCII^–^ CD11c^-^ Cx3cr1^+^ CD115^+^ Ly6C^+^ CD43^-^	5
		Macrophages	Spleen	Macrophage	iNOS	Lin^–^ CD11b^+^ F4/80^+^ CD64^+^ Ly6C^+^ CD69^+^ SCA-1^+^ MERTK^mid^ CD206^–^ MHCII^–^ CD9^–^	4
			Spleen	Macrophage	iNOS_inf		3
HUMAN TRANSCRIPTS							
GEO		Cell type	Tissue	Subtype	Sample name	Cell sorting markers	n
**GSE35449**	Beyer et al., PLOS 2012	Macrophages	PBMC	M0	M0	CD14^+^	7
			PBMC	M1	M1	CD14^+^ CD11B^Hi^ CD64^+^ CD68^+^	7
			PBMC	M2	M2	CD14^+^ CD11B^+^ CD23^+^ CD68^+^	7
**GSE107011**	Monaco et al., Cell Rep. 2019	Monocytes	PBMC	Monocyte	C_mono	CD14^+^ CD16^-^	4
		Macrophages	PBMC	Neutrophil	Neutrophils	SSC-A^High^ CD16^+^	4
		Dendritic cells	PBMC	Dendritic cell	mDC	HLA-DR^+^ CD11C^+^	4
		Lymphocytes	PBMC	B cell	B_naive	CD19^+^ CD27^-^ IgD^+^	4
			PBMC	CD4^+^ T cell	CD4_naive	CD4^+^ CCR7^+^ CD45RA^+^	4

List of data sets, which were utilized throughout this study, grouped by their gene expression omnibus (GEO) code and article reference. For each immune cell type (macrophages, dendritic cells, granulocytes, ILCs or lymphocytes) the associated immune cell subtypes or tissue-of-origin is indicated, followed by the sample name and the associated surface markers, which were used for the sorting or phenotyping of the cells. In the last column the number of biological replicate samples is mentioned for each cell type.

Differential expression was calculated using DESeq with default parameters. DESeq corrected library depth (normalization), estimated dispersions with a parametric model and fitted a negative binomial distribution to allow hypothesis testing with the Wald method. A p-value adjusted (false discovery rate) < 0.05 was considered a significant difference in expression per gene. For the sample with a single replicate, its variance was automatically calculated by DESeq2 based on the variances of the other samples (21). To generate scaled data, which was needed for some of the downstream visualizations and analyses – like principal component analysis (PCA) or Triwise plots – a version of the data sets with a corrected mean-variance relationship was calculated using the varianceStabilizingTransformation function from the DESeq2 package.

### 2.4 Analysis of microarray data set

The already normalized matrix of counts was first filtered to eliminate genes with low expression using edgeR’s filterByExpr function with a minimum CPM counts of 5 leaving 24,888 probes. After annotating the probe names with gene names we eliminated any duplications of genes by choosing the first one in order. Differential expression was calculated with the limma (v 3.54.2) (23) package by fitting a linear model in each feature using the lmFit function with default parameters followed by contrasts.fit and eBayes functions. Downstream plots were performed in the same fashion as the RNA-seq datasets.

### 2.5 Data analyses and visualization in R

PCA plots were generated with the plotPCA() function from the DESeq2 package.

The Triwise package (v 0.99.5) (24) was used to visualize differential expression on three cell types at a time in several combinations. Triwise dot plots were computed by the plotDotplot function, which transforms a three-sample expression matrix to barycentric coordinates allowing the visualization of three-dimensional expression values in a two-dimensional (2D) graph. An intuitive explanation of the rationale behind this visualization can be found in (**Supplementary Figures S1A & S1B**). Rose plots were performed with the plotRoseplot function, which makes a histogram out of the barycentric 2D expression coordinates, thus showing the number of genes in every section of the plot. R3.3 was used for the generation of these plots.

SLC genes with the highest expression per sample were chosen by taking the top 20 SLC genes with the highest mean expression across replicates for a given sample. This threshold was chosen, because the top 20 SLCs contain approximately 50% of the normalized counts of all SLCs per sample analyzed per data set. Venn diagrams showing common top SLC genes between samples were performed with the VennDiagram package (v1.7.3) (25). Heatmaps performed using the pheatmap (v1.0.12) package (26).

#### 2.5.1 Interactive dot plots

To access the six interactive dot plots (IntDP1, IntDP2, IntDP3, IntDP4, IntDP5 and IntDP6), please see the zip file in the **Supplementary Material**.

### 2.6 Data analysis in GraphPad prism

Read count comparisons from dendritic cell subsets were visualized in Prism 9 for macOS (version 9.5.0). The resulting data was analyzed using either a Mann-Whitney T test or an Ordinary one-way ANOVA with Dunnett’s multiple comparisons test.

### 2.7 Cartoon schematics

Cartoon schematics were created in part using the software from BioRender.com.

## 3 Results

### 3.1 Significantly more SLCs are expressed in phagocytes compared to lymphocytes

**Figure 1:**
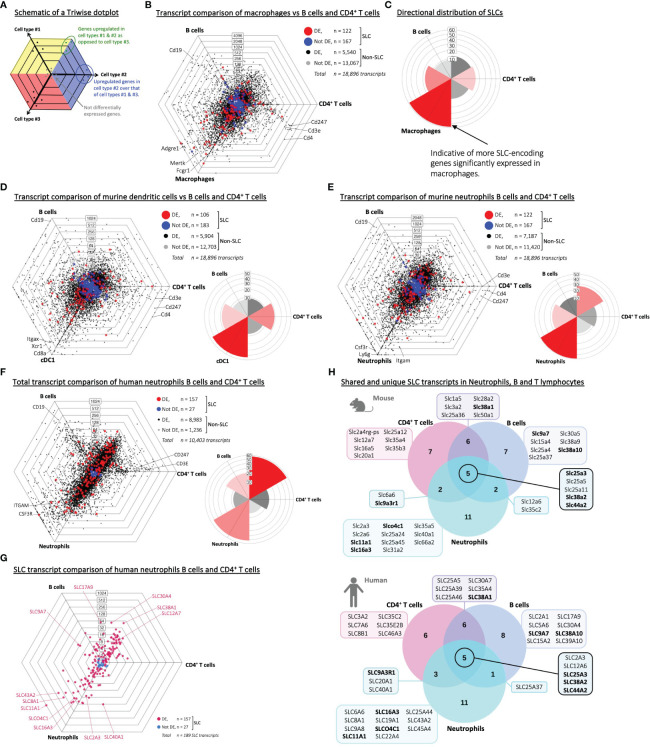
Phagocytes display a pronounced SLC transcriptome profile compared to B and T lymphocytes. **(A)** A schematic drawing of a Triwise dot plot, which explains how to interpret the gene expression comparisons between three samples or cell types. The direction of a dot shows in which cell type the gene is upregulated, while the distance from the center of the plot indicates the strength of upregulation – similar to log fold change. Genes with the same expression in all three cell types will lie close to each other in the center, regardless of the magnitude of their absolute expression levels (indicated by grey dots). The colored background in the plot (i.e., yellow, blue or red) depicts the zones, in which upregulated genes in each sample (cell type #1, #2 or #3 respectively) will be located after gene expression transformation into barycentric coordinates. When a gene is equally upregulated in two samples compared to the third sample, the dot will locate to the intersection between two zones (e.g., the green dots). **(B, C)** Triwise dot plot and rose plot of RNA-Seq data (GSE109125) from splenic immune cell subsets of the adaptive immune system (B cells and CD4^+^ T cells) versus macrophages sorted from mice. **(D, E)** Triwise comparisons and rose plots of murine cDC1 dendritic cells, B cells and CD4^+^ T cells **(D)** and between murine neutrophils, B cells and CD4^+^ T cells **(E)**. **(F)** Transcript Triwise comparison and rose plot of human PBMC-derived neutrophils, B and CD4^+^ T lymphocytes (GSE107011). **(B, D-F)** In the Triwise dot plots, genes are indicated with black dots (•) and SLC-encoding genes with red dots (•) when significantly different expressed. Genes are indicated with grey dots (•) and SLC-encoding genes with blue dots (•) when not differentially expressed. Labels on the dot plot grid lines indicate transcript fold changes (up to 4,096 folds) of reads in one cellular subset versus another. Genes encoding known cell-specific markers are indicated for each cell type: Cd19 (CD19), Cd247 (CD247), Cd3e (CD3E), Cd4 (CD4), Adgre1 (F4/80), Mertk (MER proto-oncogene, Tyrosine Kinase), Fcgr1 (Fc receptor, IgG), Itgax (CD11c), Xcr1 (XC motif chemokine Receptor 1), Cd8a (CD8 subunit Alpha), Csf3r (Colony Stimulating Factor 3 Receptor), Ly6g (Ly-6G) and Itgam (CD11b). **(C-F)** Labels on the rose plot gridlines indicate the number of genes (ranging from 10-60) included in each of the six rose petals/buckets. **(G)** Triwise comparison of SLC transcripts in human neutrophils, B cells and CD4^+^ T cells (GSE107011). Differentially expressed SLCs are depicted in magenta (•) and non-differentially expressed SLCs in light blue (•). Individual, highly and significantly different expressed SLCs are highlighted and labelled with their gene name. Labels on the dot plot grid lines indicate transcript fold changes (up to 1,024 folds) of reads in one cellular subset versus another. **(H)** Venn diagrams of the top 20 highest expressed SLCs in neutrophils, B cells and CD4^+^ T cells in mouse (upper panel) or in human (lower panel) RNA-Seq data sets (GSE109125 and GSE107011 respectively). Gene names highlighted in bold are shared between the two data sets.

During the encounter with an invading microbe or dying cell, professional phagocytes digest and turn over overwhelming amounts of cellular debris and metabolites. During this process of recycling, a large machinery of metabolite transport involving SLC proteins is necessary for the phagocyte to maintain its cell volume, intracellular pH, and inflammatory state (5). To test the premise that SLCs in general may be more expressed in phagocytes under steady state level, we made use of a three-way comparison model called Triwise, which allows us to visualize and analyze gene expression data from three biological conditions simultaneously (24) (**Figure 1A**; **Supplementary Figures S1A & S1B**). To reduce the influence of tissue-specificity on gene expression signatures, we made use of a publicly available transcriptional data set (**Table 1**) – part of the immunological genome project (ImmGen) – that contains adaptive as well as innate immune cell subsets isolated and sorted from the spleen of 6–8-week-old C57BL6/J mice (9). Our first quality control was to ensure that the samples from the respective data set formed tight clusters with little intra-sample variance compared to inter-sample variance (**Supplementary Figures S1C**). As no outliers were found on this visual inspection, we proceeded to the first comparative analysis, in which we looked at the transcriptome of macrophages compared to adaptive immune cells, specifically B lymphocytes and CD4 T lymphocytes (**Figure 1B**, Aaes et al., Interactive Dot Plots IntDP1, **Supplementary Material**). Not only did the macrophages express a significant number of genes to a higher extent than the lymphocytes, this distribution of differentially expressed genes was directly mirrored in the SLC gene family specifically (**Figure 1C**). Recent work from our lab demonstrated a transcriptional upregulation of membrane-bound transporters – especially those of the SLC3/SLC7 family – in bone marrow-derived dendritic cells during engulfment of apoptotic cells (27); to test SLC transcriptional profiles already visible at steady state, we performed a similar transcriptome comparison of type 1 conventional dendritic cells (cDC1s) to lymphocytes (**Figure 1D**, Aaes et al., Interactive Dot Plots IntDP2, **Supplementary Material**). Again, we noted an overall transcriptional distinction between dendritic cells and the adaptive immune cells, which was even more striking when evaluating the SLC gene family. A third professional phagocyte population is neutrophils, and here again we observed a significant number of differentially upregulated genes (**Figure 1E**, Aaes et al., Interactive Dot Plots IntDP3, **Supplementary Material**) and particularly of the SLCs compared to the B and T cells. To test whether these observations are also true in humans, we analyzed immune cell samples from a human RNA-Seq data set (**Supplementary Figure S1D**) (14). Similar to the corresponding murine data, the Triwise comparison of human neutrophil transcripts versus B and CD4 T lymphocytes showed a distinct gene expression signature (**Figure 1F**, Aaes et al., Interactive Dot Plots IntDP5, **Supplementary Material**), which was much pronounced in the SLC transcript subset (**Figure 1G**). Directly comparing the highest expressed SLCs, which are unique to one cell type or overlapping among two or all three immune cell types, we found several identical genes in both the human and murine data sets (**Figure 1H**). Among the hits are SLC16A3 (MCT4) – a monocarboxylate (lactate) transporter, which was recently identified as an onco-immunological biomarker and associated with increased neutrophil infiltration in tumors (28, 29), and NRAMP1, which is encoded by SLC11A1 and is highly expressed in both human as well as murine neutrophils. General to both murine and human immune cells, we also found both SLC25A3, SLC38A2 and SLC44A2 as highly expressed in both innate and adaptive immune cells. Thus, we note a pronounced SLC transcriptional profile in three distinct splenic phagocyte populations – macrophages, cDC1s, and neutrophils compared to B and CD4^+^ T lymphocytes from steady state conditions in both murine and human RNA-Seq data sets.

### 3.2 Professional phagocytes express distinct SLC transporters

**Figure 2:**
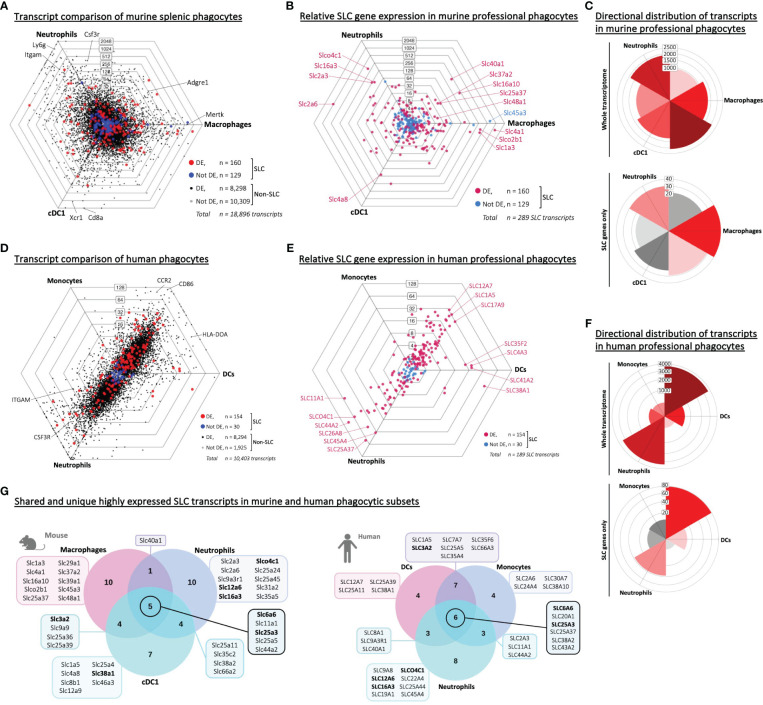
Shared and unique SLC signatures are expressed in distinct phagocyte populations from the same tissue of origin. **(A, D)** Triwise dot plot of RNA-Seq data sets representing three phagocyte populations: Murine splenic macrophages, neutrophils and cDC1 dendritic cells (GSE109125 in A) or human PBMC-derived monocytes, neutrophils and dendritic cells (GSE107011 in D). Genes are indicated with black dots (•) and SLC-encoding genes with red dots (•) when significantly different expressed. Genes are indicated with grey dots (•) and SLC-encoding genes with blue dots (•) when not differentially expressed. Labels on the dot plot grid lines indicate transcript fold changes (up to 2,048 folds) of reads in one cellular subset versus another. **(B, E)** Triwise dot plot of only the SLC-encoding genes in murine (GSE109125 in B) or human phagocytes (GSE107011 in E) indicating differentially expressed SLCs in magenta (•) and non-differentially expressed SLCs in light blue (•). Individual SLCs that are found back among the top 20 highest expressed SLCs in each cell type are highlighted and labelled. **(C, F)** Rose plots showing directional distribution of all differentially expressed genes (top panel) or the differentially expressed SLCs specifically (bottom panel) among the three phagocyte populations. The labels on the gridlines indicate the number of genes per rose petal/bucket. **(G)** The top 20 most expressed SLCs found in each type of phagocyte are compared in a Venn diagram. The gene names of the SLCs that are unique to one cell type or shared among two or all three populations are written in the boxes. Gene names in bold font are found among the top 20 most expressed SLCs in all three populations in both the murine (GSE109125) and human (GSE107011) data sets.

As all three murine phagocyte populations showed a strong upregulation of over 100 SLC-encoding genes, we queried whether these SLCs represented a general phagocyte-specific SLC profile, or whether each type of phagocyte expressed a distinct set of SLCs. Therefore, we first compared the total transcriptome of the three phagocyte populations – all originating from the spleen to minimize variables. We observed many genes that were significantly differentially expressed – including SLCs (**Figure 2A**, Aaes et al., Interactive Dot Plots IntDP4, **Supplementary Material**). We also replotted the SLCs separately (**Figure 2B**) to compare more easily the highly expressed and differentially expressed SLCs individually. The transcriptome analysis of the three types of phagocytes did not show a striking directional distribution (as seen between phagocytes and adaptive immune cells) (**Figure 2C**). We also compared the transcriptome of human phagocytic subsets (**Figure 2D**, Aaes et al., Interactive Dot Plots IntDP6, **Supplementary Material**) and plotted the SLC transcripts separately (**Figure 2E**). Unlike the ImmGen murine data, the human data set did not contain the same well defined phagocyte populations. In the human RNA-Seq data set, we could perform comparative analysis of classical monocytes (instead of murine macrophages), myeloid dendritic cells (in place of cDC1) and low-density neutrophils, which showed a directional distribution of the human transcripts, distinguishing the transcriptomes of neutrophils from monocytes and dendritic cells (**Figure 2F**). The human data set analysis again revealed similarities to the murine data, such as SLCO4C1, which in the murine data set was highly expressed in neutrophils, being abundant in human neutrophils (**Figures 2B, E**).

To test whether the phagocyte-specific differentially expressed SLCs were merely a reflection of transcriptional reads, or whether we could identify a set of highly expressed SLCs common to all phagocytes, we depicted the top 20 highest expressed SLC genes from each phagocyte in a Venn diagram and identified overlapping or uniquely highly expressed SLCs (**Figure 2G**, left panel). Five SLCs were among the top 20 in all three murine phagocytes: *Slc6a6*, a taurine transporter found in the plasma membrane; the lysosomal metal ion transporter *Slc11a1*; and three mitochondrial membrane transporters *Slc25a3*, *-5* and *Slc44a2* encoding transporters of phosphate, adenine, and choline respectively. To ensure that the SLC expression profiles were unique to phagocytes, and not just a general immune cell SLC gene profile, we compared the top 20 expressed genes of each phagocyte to adaptive immune cells (**Supplementary Figure S2A**). By comparing each of these gene lists (**Table 2**), we could identify SLC11A1 as the only transporter to be highly expressed in all three professional phagocytes, but not among the top 20 expressed SLCs in B or T lymphocytes (**Table 3**). The role of SLC11A1, also known as natural resistance-associated macrophage protein 1 or NRAMP1 in lysosomal ferrous iron transport in phagocytes has been well described in the context of anti-microbial defense (30–33). NRAMP1 is highly expressed in neutrophilic granules, which fuse with yeast *Candida albicans* containing phagosomes to deprive the microbe from nutrients as an antimicrobial defense mechanism (31), and in macrophages and dendritic cells, NRAMP1 has been linked to antigen presentation (34, 35). Although lysosomes also play an important role in T cell biology and in their release of cytotoxic molecules (36), *Slc11a1* is not expressed neither in CD4^+^ nor in CD8^+^ T lymphocytes (37). Our analysis thus revealed a high expression of SLC11A1/NRAMP1 specifically in all three phagocyte populations alluding to its multiple phagocyte-specific functions, which are different from that of lymphocytes.

**Table 2:** Unique transcripts among the most abundant SLCs in splenic phagocytes.

		Macrophages	cDC1 Dendritic cells	Neutrophils	B cells	CD4^+^ T cells
**SLCs unique to phagocytes**	Slc1a3	✗	–	–	–	–
	Slc2a3	–	–	✗	–	–
	Slc2a6	–	–	✗	–	–
	Slc4a1	✗	–	–	–	–
	Slc4a8	–	✗	–	–	–
	Slc8b1	–	✗	–	–	–
	Slc9a9	⚫	⚫	–	–	–
	Slc11a1	⚫	⚫	⚫	–	–
	Slc12a9	–	✗		–	–
	Slc16a3	–	–	✗	–	–
	Slc16a10	✗	–	–	–	–
	Slco2b1	✗	–	–	–	–
	Slco4c1	–	–	✗	–	–
	Slc25a24	–	–	✗	–	–
	Slc25a39	⚫	⚫	–	–	–
	Slc25a45	–	–	✗	–	–
	Slc29a1	✗	–	–	–	–
	Slc31a2	–	–	✗	–	–
	Slc35a5	–	–	✗	–	–
	Slc37a2	✗	–	–	–	–
	Slc39a1	✗	–	–	–	–
	Slc40a1	⚫	–	⚫	–	–
	Slc45a3	✗	–	–	–	–
	Slc46a3	–	✗	–	–	–
	Slc48a1	✗	–	–	–	–
	Slc66a2	–	⚫	⚫	–	–

The most highly expressed SLCs found in the splenic RNA-Seq data sets of adaptive immune cells (CD4^+^ T cell and B cells) versus each of the three professional phagocytes (macrophages, cDC1 and neutrophils) were compared in three separate Venn diagrams (**Supplementary Figure S1A**). The SLC genes uniquely expressed in phagocytes, and not in lymphocytes, are listed in this table. Blue shaded background and dark blue circles (⚫) indicate genes that are expressed among the top 20 SLCs in all three phagocyte populations. Light blue circles (⚫) indicate genes that are unique to two phagocyte populations, while light blue crosses (✗) indicate genes that are unique to just one phagocyte population.

**Table 3:** Shared and unique SLC transcripts in splenic phagocytes and lymphocytes.

			Macrophages	cDC1 Dendritic cells	Neutrophils	B cells	CD4^+^ T cells
**SLCs unique to lymphocytes**	**CD4^+^ T & B cells**	Slc1a5	–	–	–	⚫	⚫
		Slc3a2	–	–	–	✗	✗
		Slc25a11	–	–	–	✗	✗
		Slc25a36	–	–	–	✗	✗
		Slc28a2	–	–	–	⚫	⚫
		Slc38a1	–	–	–	⚫	⚫
		Slc38a2	–	–	–	✗	✗
		Slc50a1	–	–	–	⚫	⚫
	**CD4^+^ T cells**	Slc2a4rg-ps	–	–	–	–	⚫
		Slc9a3r1	–	–	–	–	⚫
		Slc12a7	–	–	–	–	⚫
		Slc16a5	–	–	–	–	⚫
		Slc20a1	–	–	–	–	⚫
		Slc25a12	–	–	–	–	⚫
		Slc35a4	–	–	–	–	⚫
		Slc35b3	–	–	–	–	⚫
	**B cells**	Slc9a7	–	–	–	⚫	–
		Slc12a6	–	–	–	⚫	–
		Slc15a4	–	–	–	⚫	–
		Slc25a4	–	–	–	⚫	–
		Slc25a37	–	–	–	⚫	–
		Slc30a5	–	–	–	⚫	–
		Slc35c2	–	–	–	✗	–
		Slc38a9	–	–	–	⚫	–
		Slc38a10	–	–	–	⚫	–
**SLCs shared between phagocytes & lymphocytes**	**CD4^+^ T & B cells**	Slc1a5	–	✗	–	✗	✗
		Slc3a2	⚫	⚫	–	⚫	⚫
		Slc25a3	⚫	⚫	⚫	⚫	⚫
		Slc25a5	⚫	⚫	⚫	⚫	⚫
		Slc25a11	–	⚫	⚫	⚫	⚫
		Slc25a36	–	✗	–	✗	✗
		Slc38a1	–	✗	–	✗	✗
		Slc38a2	–	⚫	⚫	⚫	⚫
		Slc44a2	⚫	⚫	⚫	⚫	⚫
	**CD4^+^ T cells**	Slc6a6	⚫	⚫	⚫	–	⚫
		Slc9a3r1	–	–	✗	–	✗
	**B cells**	Slc12a6	–	–	✗	✗	–
		Slc25a4	–	✗	–	✗	–
		Slc25a37	✗	–	–	✗	–
		Slc35c2	–	⚫	⚫	⚫	–

The top 20 expressed SLCs in the splenic RNA-Seq data sets of lymphocytes versus professional phagocytes were compared in three separate Venn diagrams (**Supplementary Figure S1A**). Dark blue circles (⚫) on dark background are SLCs unique to lymphocytes in all three phagocyte comparisons. SLCs uniquely expressed in lymphocytes in two phagocyte comparisons are shown by light blue circles (⚫), and if one comparison with light blue crosses (✗). SLCs on a shaded red background and dark red circles (⚫) are highly expressed in lymphocytes as well as in all three phagocytes. Bright red circles (⚫) are SLCs shared among two phagocytes and lymphocytes, while bright red crosses (✗) indicate SLCs that are shared between just one phagocyte and lymphocytes.

This analysis also revealed highly expressed SLCs unique to one of the lymphocyte populations (**Table 3**); interestingly, *Slc6a6*, *Slc25a3*, *Slc25a5* and *Slc44a2* were generally high in B lymphocytes and/or CD4 T cells. SLC6A6 has been described for its role in T cell immunity, in which a high expression is positively associated with CD8 effector T cell proliferation and function (38), while in macrophages, SLC6A6 is found upregulated during polarization to a more pro-inflammatory phenotype (39). To our knowledge, no immune cell-specific role of the three mitochondrial transporters has yet been described, and this may lead to the exploration of new research domains for their transport functions in innate and adaptive immunity.

Finally, we compared the top 20 most expressed SLCs in phagocytes across the human and murine data sets (**Figure 2G**). We found a number of SLCs that are similarly expressed - despite the difference in immune cell host (human vs mouse), tissue (peripheral blood vs spleen) and immune cell subtypes (monocyte vs macrophage; myeloid DC vs cDC1). Thus, even based on just these two data sets, we could draw parallels between SLC gene expression patterns in human and murine immune cell subsets, and this data analysis points toward a role for specific SLCs as potential novel immune cell markers.

To examine the SLC transcripts in other innate immune cell subsets, we compared the total transcriptome and the SLC genes in innate lymphoid cell (ILC) subsets, namely ILC1-like NK cells, ILC2 and ILC3 (**Supplementary Figure S2B**). Although these cells were isolated from the spleen or intestinal lamina propria, the directional distribution of differentially expressed genes of the total transcriptome and SLC family were evenly spread across the three ILC populations. When narrowing the analysis to only ILC2 and ILC3 subsets of the small intestine, a clear directional distribution of differentially expressed genes was seen between the two types of lymphoid cells; while 243 out of 289 of the SLC transcripts were not differentially expressed, a few distinct SLCs appeared highly upregulated in one over the other populations, such as the calcium-dependent L-proline and glycine importer SLC6A20 in pro-inflammatory NKp46^+^ ILC3 cells and confirmed the ILC2-specificity of the large amino acid transporter SLC7A8 (40, 41) (**Supplementary Figure S2C**). Thus, through these three-way transcriptome comparisons of innate and adaptive immune cell subsets, we identify known and novel SLC signatures that are shared or unique to multiple cell types.

### 3.3 Macrophage-specific SLCs in tissue sites or differentiation states

**Figure 3:**
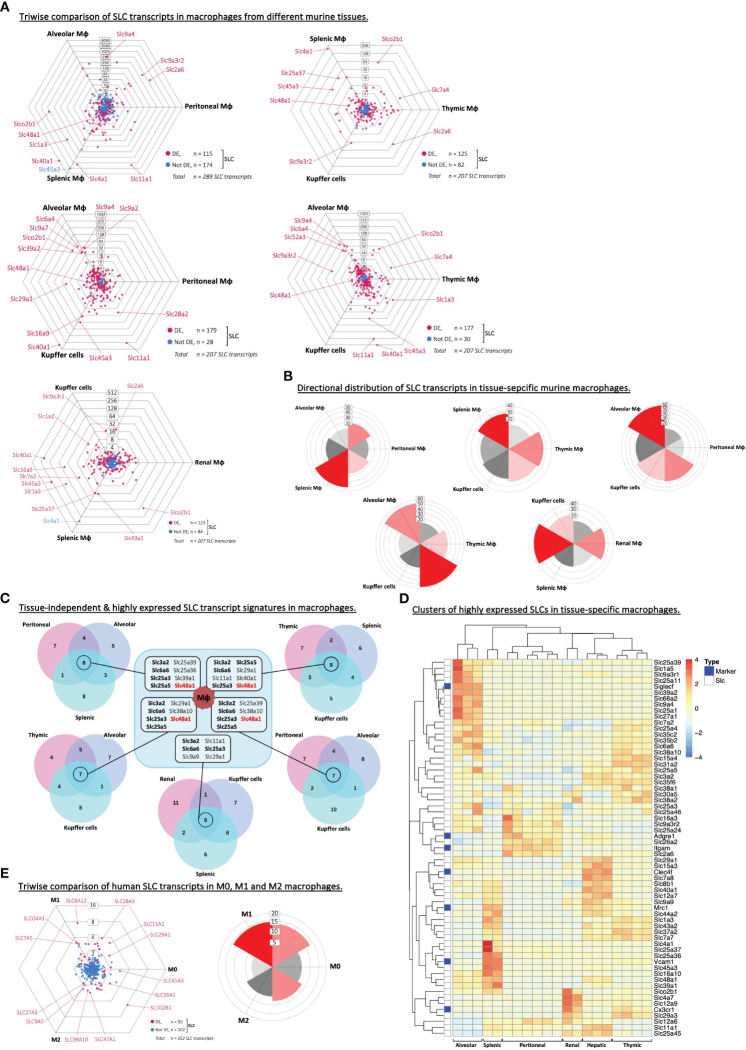
Macrophages express unique SLCs independent of the tissue of origin. **(A)** Triwise dot plots of the SLC-encoding genes in RNA-Seq data sets from alveolar, peritoneal and splenic macrophages (GSE109125), or from Kupffer cells, splenic, thymic, renal, alveolar or peritoneal macrophages (GSE122108). Differentially expressed SLCs are depicted in magenta (•) and non-differentially expressed SLCs in light blue (•). Individual, highly and significantly different expressed SLCs are highlighted and labelled with their gene name. Labels on the dot plot grid lines indicate transcript fold changes (up to 4,096 folds) of reads in one cellular subset versus another. **(B)** Rose plots indicating the directional distribution of all differentially expressed SLCs in each Triwise comparison respectively. Gridline labels indicate the number of genes per rose petal/bucket. **(C)** The top 20 most expressed SLCs found in each macrophage subtype are compared in a Venn diagram corresponding to each Triwise dot plot and rose plot. The gene names of the SLCs that are shared among all three macrophage subsets are outlined in the box in the middle. Gene names in bold font are also among the top 20 highest expressed SLCs in adaptive immune cells. Gene names in red bold font are unique to macrophages and not found among the top 20 most expressed SLCs in other phagocytes, nor in B or CD4^+^ T lymphocytes. **(D)** The reads of the top 20 most expressed SLCs in RNA-Seq data sets (GSE122108) from six macrophage subsets were compared in a heat-map. Clusters of highly expressed SLCs, representative of each macrophage subtype, are indicated on the y axis, while clusters of tissue origin are indicated on the x axis. The color grading in the heat map ranging from blue to red indicates the relative gene expression. Genes encoding known macrophage-specific markers are highlighted with dark blue boxes along the y axis: Siglecf (Sialic Acid Binding Ig Like Lectin F), Cx3cr1 (C-X3-C Motif Chemokine Receptor 1), Adgre1 (F4/80), Itgam (CD11b), Mrc1 (Mannose Receptor C-Type 1), Clec4f (C-Type Lectin Domain Family 4 Member F) and Vcam1 (Vascular Cell Adhesion Molecule 1). **(E)** Triwise comparison and rose plot of the directional distribution of SLC transcripts among human M0, M1 and M2 macrophages (GSE35449).

Our analyses so far indicated that each type of phagocyte expressed a set of highly abundant SLC transcripts, which were not among the top 20 SLCs in lymphocytes. To assess whether the tissue of origin may impact these SLC gene lists, we expanded our analysis to include other subsets or tissue-specific phagocytes. First, we compared the SLC transcriptome of macrophages from six different tissues, and two independent murine RNA-Seq data sets (**Figure 3A**; **Table 1**; **Supplementary Figure S1E**). This revealed a large fraction (from 40% to 86%) of SLCs that are differentially expressed between tissue-specific macrophages. The differentially expressed SLCs showed a preferred polarization toward alveolar, splenic and renal macrophages (**Figure 3B**), which may indicate a higher turnover of metabolites in these tissues, especially in the kidney-derived macrophages; as for the other tissues, the directional distribution of SLCs was analogous to that of the entire transcriptome (**Supplementary Figure S3A**). More specifically, alveolar macrophages express a significantly higher level of several Na^+^/H^+^ exchangers (i.e. NHE) belonging to the SLC9 family, while transporters of divalent cation – e.g. ferrous iron (Fe^2+^) – such as SLC11A1 (i.e. NRAMP1) and SLC40A1 (i.e. Ferroportin) as well as a known heme transporter SLC48A1 (i.e. HRG1) were expressed at significantly higher levels in macrophages isolated from tissues of high red blood cell turnover (i.e. erythrophagocytosis), such as spleen and liver-resident macrophages (Kupffer cells). As an attempt to identify a common highly expressed SLC gene signature specific to macrophages independent of their tissue of origin, we compared the overlapping genes among the top 20 SLCs in each of the five macrophage subset comparisons (**Figure 3C**). Aside from the four SLCs (*Slc3a2*, *Slc6a6*, *Slc25a3*, *-5*), which were found to be highly expressed also in adaptive immune cells (**Table 3**), we could identify *Slc48a1* as a highly expressed SLC common to all but one macrophage subsets studied. Interestingly, it has previously been demonstrated in bone marrow-derived macrophages, that HRG1 colocalizes with NRAMP1 (SLC11A1) on phagolysosomal membranes containing ingested senescent red blood cells (RBCs) (42), once again suggesting the importance of iron and heme transport in erythrophagocytosis. Finally, heatmap analyses of the top 20 expressed SLCs within each tissue-specific macrophage subset revealed clear clusters that often correlated with tissue-specific markers such as Siglec-F associated with alveolar macrophages and CLEC4F specific for Kupffer cells (**Figure 3D**; **Supplementary Figure S3B**). Thus, we could identify tissue-specific SLC signatures on macrophages, and identified a shared (i.e., tissue independent) SLC marker, SLC48A1/HRG1.

Besides tissue-specificity, we also examined if macrophages under different differential states would express specific SLCs (**Table 1**). The human microarray data set allowed us to compare M0, M1 and M2 macrophages derived from seven healthy human donors (20). Although the current view is that this M1 vs M2 categorization is too rigid and that *in vivo* it is to be more fluid or plastic with M1-like and M2-like phenotypes that can further change, we used the existing data sets to get a gauge on SLC expression in macrophages depending on their differentiation states. The variance between the three cell types was very low (**Supplementary Figure S1F**) and the difference in overall transcript expressions was also markedly less pronounced (**Supplementary Figure S3C**). Comparing the top 20 highest expressed SLCs in each of the three cell types, the majority of transcripts was shared among all three, while the M0 macrophages expressed no unique SLCs (**Supplementary Figure S3D**). Upon looking more specifically at the SLC transcripts, we observed mild but significant differences between a few SLC genes, which may point toward a role in macrophage differentiation or their function (**Figure 3E**).

### 3.4 SLC expression in dendritic cell subsets

**Figure 4:**
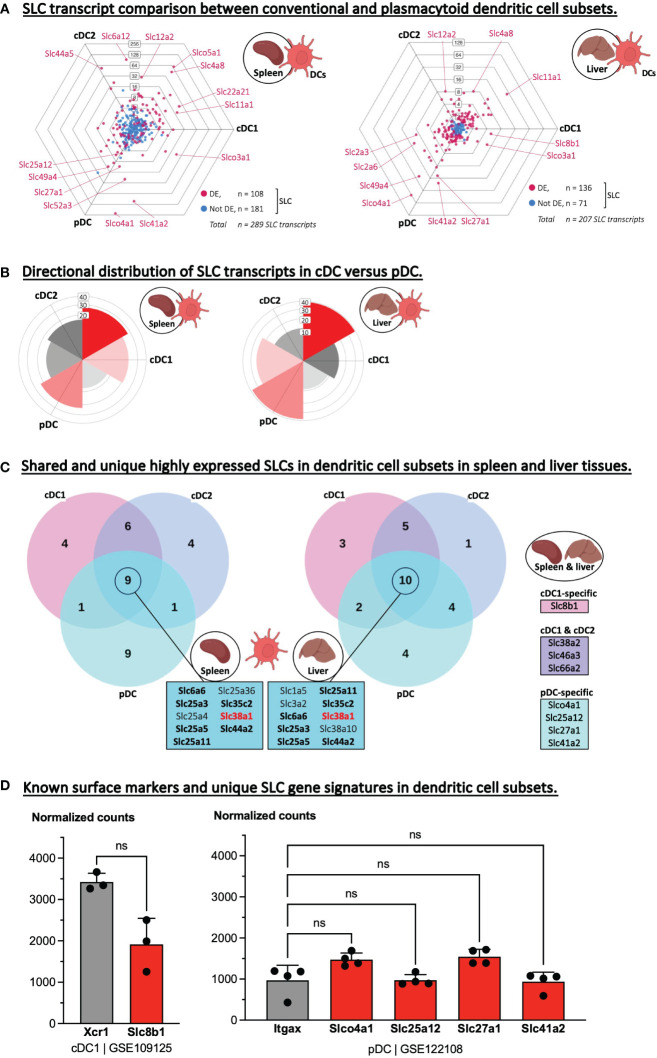
Identifying highly expressed SLCs as potential new markers of dendritic cells subsets. **(A)** Triwise dot plots of the SLC-encoding genes in RNA-Seq data sets representing conventional dendritic cell type 1 and 2 (cDC1 and cDC2) as well as plasmacytoid DCs deriving from the spleen (GSE109125, left) or isolated from the liver (GSE122108, right). Differentially expressed SLCs are depicted in magenta (•) and non-differentially expressed SLCs in light blue (•). Individual, highly and significantly different expressed SLCs are highlighted and labelled with their gene name. Labels on the dot plot grid lines indicate transcript fold changes (up to 256 folds) of reads in one cellular subset versus another. **(B)** Rose plots indicating the directional distribution of all differentially expressed SLCs in each Triwise comparison respectively. Gridline labels indicate the number of SLCs per rose petal/bucket. **(C)** The top 20 most expressed SLCs found in each dendritic cell subtype are compared in a Venn diagram corresponding to the two Triwise dot plots. The gene names of the SLCs that are shared among all three DC subsets are outlined in the boxes in the middle. Gene names in **bold font** are also among the top 20 highest expressed SLCs in adaptive immune cells. Gene names in **red bold font** are unique to DCs and not found among the top 20 most expressed SLCs in other phagocytes, nor in B or CD4^+^ T lymphocytes. Highly expressed SLCs, true to both the splenic and hepatic comparisons, are indicated in boxes (right panel) and represent SLCs uniquely expressed in cDC1 subsets, pDC subsets or shared among the most expressed SLCs in cDC1 & cDC2 subsets. **(D)** Normalized read counts of uniquely expressed SLCs specific for cDC1 or to pDCs were compared to a known cDC1 subset-specific marker, Xcr1 (encoding XC motif chemokine Receptor 1) or to a general DC-specific marker, Itgax (encoding CD11c), respectively. Each dot represents one biological replicate (= cells isolated from one mouse) and are plotted as the mean with standard deviation as error bars. The cDC1 gene set was analyzed with a Mann-Whitney, two-tailed T test. The pDC gene set was analyzed with a one-way ANOVA, Dunnett’s multiple comparisons test; Itgax served as control; ns, not significant.

Next, we examined the transcriptome of dendritic cell subsets isolated from two different tissues. We limited this analysis to the main conventional dendritic cell (cDC) subsets, cDC1 and cDC2, and plasmacytoid dendritic cells (pDC). A three-way comparison of the total transcriptome confirmed a clear difference in the overall gene expression between cDCs and pDCs (**Supplementary Figure S4A**), and strikingly, this difference became even more noticeable when we plotted only the SLC gene sets (**Figures 4A, B**). Interestingly, some of the same SLCs were significantly upregulated in one DC subtype over the other in both the splenic as well as in the hepatic data set e.g., *Slc11a1* in cDC1, *Slc12a2* in cDC2, and *Slco4c1* and *Slc41a2* in pDC. Therefore, we aimed to identify SLCs that are unique to one DC subset or general to all three subsets by comparing the highest expressed SLCs of each subset with each other and across the two tissues (**Figure 4C**). This analysis resulted in nine and ten overlapping SLCs in the spleen and liver data set respectively, and upon comparing the two intersecting SLC gene sets, seven SLCs were identical, of which just one transporter, *Slc38a1*, was unique to dendritic cells and not appearing in the other phagocyte or adaptive immune cell comparisons (**Table 2**). SLC38A1, or SNAT1, is a sodium-dependent importer of Glutamine, and is reported in neurons to be expressed in the somatodendritic compartment, hinting towards a role in dendritic cell plasticity (43). We further compared the tissue-overlapping, highly expressed SLCs, which were unique to one or two dendritic cell subsets. *Slc8b1* encodes a mitochondrial sodium/calcium exchanger protein and was found uniquely in the cDC1 subset. Similarly, a gene set of four SLCs were unique to pDCs, while three other SLCs were highly expressed exclusively in the two conventional DC subsets. If these various SLC transcripts were indeed highly abundant also compared to other genes, then they could act as potential new markers of DC subsets. Hence, we plotted and tested the expression levels of each potential SLC marker against known DC markers such as XCR1 in cDC, *Sirpa* encoding CD172a/SH2 in cDC2 and *Itgax* encoding the general DC marker, CD11c, in pDCs (**Figure 4D**; **Supplementary Figure S4B**). In the spleen, *Slc8b1* (i.e., NCLX) transcript levels were not different from that of XCR1, which is a highly specific marker and chemokine receptor on cDC1. To our knowledge, no DC-specific role of SLC8B1 has been described to date, but mitochondrial calcium fluxes have been linked to phagocytosis, phagosomal ROS production and pathogen killing by macrophages (44).

The four potential pDC-specific SLCs were not differentially expressed from CD11c (encoded by *Itgax*) – a general DC marker – and they are therefore less likely to be used as pDC-specific markers (**Figure 4D**; **Supplementary Figure S4B**). Of the shared cDC1 and cDC2 markers, the glutamine importer encoded by *Slc38a2* especially caught our attention, because very recently, SLC38A2 and glutamine signaling specifically in cDC1s was shown to dictate anti-tumor immunity *in vivo* (45). Since our analysis identified two sodium-coupled neutral amino acid transporters (SNAT) encoded by *Slc38a1* and *Slc38a2* as possible markers of all DCs or of conventional DCs, respectively, it will be interesting to expand the latest findings on SLC38A2 function in cDC1 and anti-tumor immunity (45) to other disease models and to cDC2s.

In conclusion, dendritic cell subsets isolated from two different tissues, spleen and liver, showed similar total and SLC-specific directional transcriptome distributions, and we could identify highly expressed SLCs that may act as (intracellular) markers of cDC1-specific, pDC-specific or of both cDC1 and -2.

### 3.5 SLC expression in neutrophils

**Figure 5:**
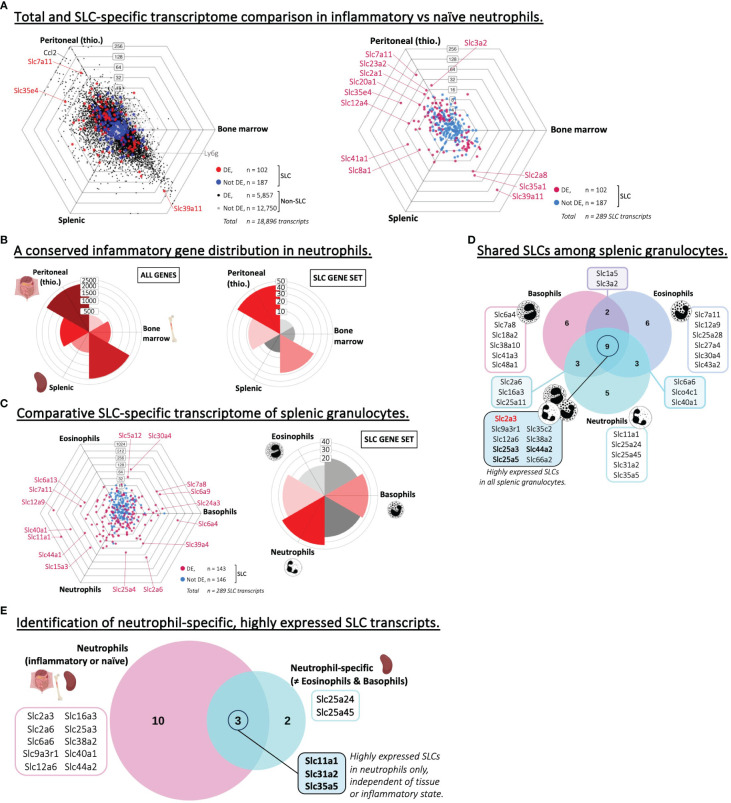
Neutrophils express specific SLCs different from other types of granulocytes. **(A)** Triwise dot plot of RNA-Seq data sets representing neutrophil subsets isolated from the peritoneum after thioglycolate-induced peritonitis, from the bone marrow or the spleen (GSE109125). In the left panel, genes are indicated with black dots (•) and SLC-encoding genes with red dots (•) when significantly different expressed. Genes are indicated with grey dots (•) and SLC-encoding genes with blue dots (•) when not differentially expressed. In the right panel, differentially expressed SLCs are depicted in magenta (•) and non-differentially expressed SLCs in light blue (•). Individual SLCs in each neutrophil subset are highlighted and two neutrophil-specific markers are labelled with their gene name: Ccl2 (encoding C-C Motif Chemokine Ligand 2) and Ly6g (Ly-6G). Labels on the dot plot grid lines indicate transcript fold changes (up to 256 folds) of reads in one cellular subset versus another. **(B)** Rose plots showing the directional distribution of all differentially expressed genes (left panel) or the differentially expressed SLCs specifically (right panel) among the three neutrophil subsets. The labels on the gridlines indicate the number of genes per rose petal/bucket. **(C)** Triwise dot plot (left panel) comparing the transcription level of SLC-encoding genes in RNA-Seq data sets representing three splenic granulocyte subsets: eosinophils, basophils and neutrophils (GSE109125). Differentially expressed SLCs are depicted in magenta (•) and non-differentially expressed SLCs in light blue (•). Individual, highly and significantly different expressed SLCs are highlighted and labelled with their gene name. Labels on the dot plot grid lines indicate transcript fold changes (up to 1,024 folds) of reads in one cellular subset versus another. Rose plot (right panel) indicating the directional distribution of all differentially expressed SLCs among the three granulocyte subsets. Gridline labels indicate the number of SLCs per rose petal/bucket. **(D)** The 20 most expressed SLCs found in each granulocyte subset are compared in a Venn diagram. The gene names of the SLCs that are unique to one subset or shared among two or all three granulocyte populations are written in the boxes. Gene names in **bold font** are also among the top 20 highest expressed SLCs in other phagocytes and adaptive immune cells. Gene names in **red bold font** are unique to granulocytes and not found among the top 20 most expressed SLCs in other phagocytes, nor in B or CD4^+^ T lymphocytes. **(E)** Highly expressed SLCs in neutrophils regardless of their inflammatory state (identified in **Supplementary Figure S4C**) were compared to those specifically expressed in neutrophils and not in other granulocyte subsets **(D)**. Unique or overlapping genes are written out in the associated boxes.

Next, we analyzed and compared the transcriptome of neutrophils isolated from either inflamed (thioglycolate-induced) peritoneal cavities or steady state neutrophils from either bone marrow or spleen (**Figure 5A**). The total gene sets showed a clear difference between inflamed and healthy tissues. This polarized, inflammation-induced gene expression signature was also clear when comparing the directional distribution of differentially expressed genes and that of SLCs specifically (**Figure 5B**), as a significant number of differentially expressed SLCs were either upregulated or downregulated specifically in inflammatory neutrophils compared to the two naïve subsets. A link between SLCs and inflammation has previously been described in the context of amino acid SLC transporters of the SLC1 and SLC7 families (46), and might explain why we observe significantly increased expression of heteromeric amino acid transporter complex SLC3A2/SLC7A11 in the inflamed tissue of our analysis. This observation also nicely coincides with recent findings from our lab, showing that SLC7A11 is highly upregulated in innate immune cells of inflamed skin (27).

To compare the SLC signature in neutrophils to other granulocyte subsets, we performed an additional comparison between eosinophils, basophils and neutrophils (**Figure 5C**; **Supplementary Figures S5A, B**). We found SLC transcripts that were upregulated in one or several of the three granulocyte populations, with a slight preference towards basophils and neutrophils over that of eosinophils. Also, in the three granulocyte populations, we could detect a list of common highly expressed SLCs – nine in total – of which the glutamine transporter GLUT3, encoded by *Slc2a3*, was the only transcript not to be associated with any other phagocyte or adaptive immune cell expression profile (**Figure 5D**; **Table 2**). Neutrophils from colorectal tumor sites have been correlated with GLUT3 expression (47), and upon neutrophil activation e.g. by bacteria, GLUT3 moves from the intracellular environment to the plasma membrane (48). Further, we wanted to identify potential neutrophil-specific SLC markers, which were not associated with a high expression in other granulocytes, and which were highly expressed independent of the tissue of origin and the inflammatory state. Therefore, we compared the three neutrophil subsets isolated from inflamed peritoneum, or steady state bone marrow or spleen (**Supplementary Figure S5C**). By overlaying the 13 SLC-encoding genes found in all three neutrophil populations, we identified five neutrophil-specific SLCs, which were not found in other granulocytes. Further, we could identify three SLCs that are highly expressed specifically in neutrophils, independent of the tissue or inflammatory state (**Figure 5E**). Of these three genes, we know now that *Slc11a1*, which encodes NRAMP1, is generally highly expressed in all phagocytic cells (**Table 2**). SLC31A2 is involved in copper uptake across intracellular membranes and has been associated with high expression in atherosclerotic plaques, which are linked with neutrophil degranulation (49). The function of SLC35A5 is predicted to be related to UDP-sugar transport, but otherwise this is an orphan transporter with no obvious function. Based on this analysis, addressing SLC35A5 in neutrophils may be worthy of pursuit.

We also investigated whether infections may change the SLC transcriptome of phagocytes. For this purpose, we found an RNA-Seq data set based on monocyte or macrophage samples from naïve or *Salmonella*-infected mice (13) (**Table 1**). Our analyses showed that bystander and infected macrophages were clearly distinguishable from their monocyte ancestors (**Supplementary Figure S1F**). This difference was visible among the total transcript distribution (**Supplementary Figure S5D**) as well among the SLC transcript distribution (**Supplementary Figure S5E**). Interestingly, when comparing the top 20 highest expressed SLCs in each of the three samples, there was a majority of SLCs shared among the three – perhaps due to their shared monocyte ancestor – while the bystander and infected macrophages shared especially one highly expressed SLC, namely *Slc48a1* or HRG1, which we earlier identified as a common SLC in macrophages of various tissue of origin (**Supplementary Figure S5F**).

## 4 Discussion

The SLC superfamily is the largest group of membrane transporters and is growing with the identification of new members based on both primary and tertiary sequence similarities and functional studies. Many SLCs are still ‘orphan transporters’ with respect to their substrate, subcellular location or method of transport. Work from our lab and others have identified specific SLC transporters to play important roles during clearance of apoptotic cells (efferocytosis) and in anti-microbial defense. However, a more systematic and focused approach to elucidate the role of SLCs in phagocytes is still needed. With this work, we sought to: (1) identify SLCs that were unique to phagocytes, (2) identify SLCs that are unique to phagocytes subsets in specific tissues and contexts/inflammation, and (3) identify SLCs that may serve as new immune cell-specific markers. In our first analysis, we compared the transcriptome of three professional phagocyte populations to that of B and CD4^+^ T lymphocyte subsets. This revealed a significant increase in SLC transcript expression in the phagocytic innate immune cell compartment compared to the lymphocytes, which represented the adaptive immune cells. Further, there were also highly expressed SLCs specific to the lymphocyte subsets, and future research is needed on these transporters in B and CD4 T lymphocytes.

General to the innate and adaptive immune cell populations, on which the analyses throughout this work were based, we were able to identify a list of four common and highly expressed SLCs: SLC6A6, SLC25A3, SLC25A5 and SLC44A2. Besides the known role of taurine transport by SLC6A6 in T cell immunity and polarization of inflammatory macrophages (38, 39), an immunity-specific role of the additional three mitochondrial transporters has not been demonstrated.

### 4.1 Iron and heme transporters are highly expressed specifically in phagocytes

Our analyses revealed a tendency of increased expression of an importer of divalent cations (SLC11A1), which contributes to recycling of iron as well as a heme transporter (SLC48A1) in phagocytic populations. Besides the known role of especially SLC11A1/NRAMP1 in phagocytic anti-microbial defense (30–33), iron fluxes have also been linked to apoptotic cell clearance – or more specifically to erythrophagocytosis. A homologue or SLC11A1 and second member of the SLC11 family member, SLC11A2 (also known as NRAMP2/DMT1) has been described for its role in ferrous iron import in phagolysosomal membranes of macrophages during erythrophagocytosis (50). However, in our interactive plots comparing the transcriptome of phagocytic populations to those of lymphocytes, *Slc11a2* locates to the very middle, and does not show the same phagocyte-specific expression as *Slc11a1*.

Related to erythrophagocytosis, HRG1, which is encoded by *Slc48a1*, was the single SLC we found to be highly expressed and unique to macrophages from five distinct tissues as well as in bystander or infected macrophages from *Salmonella*-infected mice. SLC48A1 is known to mediate heme export from phagolysosomes following RBC ingestion (51), which may explain its high expression in macrophages isolated from the bone marrow, liver and spleen – sites of high RBC turnover; however, SLC48A1 function in lung macrophages, peritoneal macrophages and its association to macrophages during infection with *Salmonella* remain to be defined.

Special attention must be paid to transcriptome analyses (e.g. RNA-Seq) of primary phagocytes, since we know from our own data (5), and that of others (52) that RNA contamination from engulfed and ingested cellular material inside phagocytes may complicate the downstream transcriptome analysis. In our analysis of the ImmGen data sets, we found that *Slc4a1* expression was highly associated with macrophages. While this chloride/bicarbonate exchanger may function in phagocytes, it was initially characterized in erythrocytes as Band3, which increases the blood’s capacity to carry carbon dioxide as plasma bicarbonate (53). Some of the phagocyte populations analyzed were from the splenic red pulp, an area where the macrophages could have engulfed erythrocytes as part of the homeostatic RBC turnover (54). While mature erythrocytes were long thought not to have mature mRNA (55), macrophages could have acquired the *Slc4a1* mRNA from reticulocyte remnants, and this remains to be determined.

### 4.2 SLCs as expression markers of DC subsets

Specific to cDC1 dendritic cells, our analysis identified a highly expressed mitochondrial sodium/calcium exchanger (NCLX) encoded by *Slc8b1*. In fact, in one data set, the expression level of *Slc8b1* was even at the level of *Xcr1*, which is a known marker of cDC1. SLC8B1 is highly expressed in mitochondrial cristae, where it conducts sodium-dependent calcium efflux (56). Mitochondrial calcium overload contributes to neurodegenerative diseases (57) as it was demonstrated in a neuronal-specific deletion model of SLC8B1 in mice (58), however, to our knowledge, no DC-specific role of SLC8B1 has yet been described. Previously, calcium fluxes in phagocytes were found to be linked to apoptotic cell engulfment as demonstrated in *C. elegans* (59), where the knockdown of two genes encoding calcium influx transporters in the plasma membrane resulted in decreased apoptotic cell removal. In mice and in murine macrophages, mitochondrial calcium fluxes and signaling are also tightly regulated with phagocytosis. Upon pathogen killing, murine macrophages rapidly increase their cytosolic calcium followed by activation of a calcium uniporter, MCU, which ensures a rapid influx of calcium into the mitochondrial matrix (44). The mitochondrial influx of calcium activates a metabolic switch, which aids in phagosomal ROS production and hence pathogen killing. One could hypothesize that SLC8B1 serves as a brake on this mechanism, and that its knockdown would increase the antimicrobial activity of cDC1 cells.

### 4.3 Neutrophils upregulate a specific SLC signature in response to induced inflammation

Throughout this manuscript, we based our results on immune cells isolated from mice that were untreated and hence the transcriptomes represented in general the homeostatic gene expression signatures. However, in one analysis we compared neutrophils isolated from untreated versus thioglycolate-injected mice – i.e., a model of induced peritonitis. Not only do neutrophils, from the inflamed mice clearly distinguish themselves from that of the untreated mice, this skew was also markedly present in the analysis of just the SLC superfamily of genes. In the inflammatory neutrophils, both *Slc2a1* (encoding Glucose transporter 1; GLUT1) and *Slc23a2* (encoding Sodium-dependent vitamin C transporter 2; SVCT2) were highly upregulated compared to the non-inflamed counterparts. Interestingly, a recent paper demonstrated for the first time a direct transport pathway between SLC23A2 and SLC2A1 of ascorbic acid (aka Vitamin C) and its connection to tooth formation in rats during wound healing (60). Thus, our observations support the view of neutrophils as important players in wound healing and tissue repair (61) and highlight SLC transporters as possible key players in this process.

## Data availability statement

Publicly available datasets were analyzed in this study. This data can be found here: GEO accession: GSE109125 ImmGen ULI: Systemwide RNA-seq profiles (#1) https://www.ncbi.nlm.nih.gov/geo/query/acc.cgi?acc=GSE109125 GEO accession: GSE122108 ImmGen ULI: OpenSource Mononuclear Phagocytes Project https://www.ncbi.nlm.nih.gov/geo/query/acc.cgi?acc=GSE122108 GEO accession: GSE164255 Hoffman et al., Immunity 2021 https://www.ncbi.nlm.nih.gov/geo/query/acc.cgi?acc=GSE164255 GEO accession: GSE35449 Beyer et al., PLOS One 2012 https://www.ncbi.nlm.nih.gov/geo/query/acc.cgi?acc=GSE35449 GEO accession: GSE107011 Monaco et al., Cell Rep. 2019 https://www.ncbi.nlm.nih.gov/geo/query/acc.cgi?acc=GSE107011.

## Author contributions

TA: Conceptualization, Formal Analysis, Funding acquisition, Investigation, Methodology, Visualization, Writing – original draft, Writing – review & editing. JC: Data curation, Methodology, Software, Visualization, Writing – review & editing. KR: Conceptualization, Funding acquisition, Project administration, Supervision, Writing – review & editing.

## References

[1] Seal RL Braschi B Gray K Jones TEM Tweedie S Haim-Vilmovsky L. Genenames.org: the HGNC resources in 2023. Nucleic Acids Res (2023) 51(D1):D1003–D9. doi: 10.1093/nar/gkac888 PMC9825485 36243972

[2] Schaller L Lauschke VM. The genetic landscape of the human solute carrier (SLC) transporter superfamily. Hum Genet (2019) 138(11-12):1359–77. doi: 10.1007/s00439-019-02081-x PMC6874521 31679053

[3] César-Razquin A Snijder B Frappier-Brinton T Isserlin R Gyimesi G Bai X. A call for systematic research on solute carriers. Cell (2015) 162(3):478–87. doi: 10.1016/j.cell.2015.07.022 26232220

[4] Freemerman AJ Zhao L Pingili AK Teng B Cozzo AJ Fuller AM. Myeloid slc2a1-deficient murine model revealed macrophage activation and metabolic phenotype are fueled by GLUT1. J Immunol (2019) 202(4):1265–86. doi: 10.4049/jimmunol.1800002 PMC6360258 30659108

[5] Morioka S Perry JSA Raymond MH Medina CB Zhu Y Zhao L. Efferocytosis induces a novel SLC program to promote glucose uptake and lactate release. Nature (2018) 563(7733):714–8. doi: 10.1038/s41586-018-0735-5 PMC6331005 30464343

[6] Perry JSA Morioka S Medina CB Iker Etchegaray J Barron B Raymond MH. Interpreting an apoptotic corpse as anti-inflammatory involves a chloride sensing pathway. Nat Cell Biol (2019) 21(12):1532–43. doi: 10.1038/s41556-019-0431-1 PMC7140761 31792382

[7] Price CTD Abu Kwaik Y. The transcriptome of legionella pneumophila-infected human monocyte-derived macrophages. PloS One (2014) 9(12):e114914. doi: 10.1371/journal.pone.0114914 25485627 PMC4259488

[8] Barrett T Wilhite SE Ledoux P Evangelista C Kim IF Tomashevsky M. NCBI GEO: archive for functional genomics data sets—update. Nucleic Acids Res (2013) 41(D1):D991–D5. doi: 10.1093/nar/gks1193 PMC3531084 23193258

[9] Heng TSP Painter MW Elpek K Lukacs-Kornek V Mauermann N Turley SJ. The Immunological Genome Project: networks of gene expression in immune cells. Nat Immunol (2008) 9(10):1091–4. doi: 10.1038/ni1008-1091 18800157

[10] Yoshida H Lareau CA Ramirez RN Rose SA Maier B Wroblewska A. The cis-regulatory atlas of the mouse immune system. Cell (2019) 176(4):897–912.e20. doi: 10.1016/j.cell.2018.12.036 30686579 PMC6785993

[11] Gainullina A Mogilenko DA Huang L-H Todorov H Narang V Kim K-W. Network analysis of large-scale ImmGen and Tabula Muris datasets highlights metabolic diversity of tissue mononuclear phagocytes. Cell Rep (2023) 42(2):112046. doi: 10.1016/j.celrep.2023.112046 36708514 PMC10372199

[12] Benoist C the ImmGen C. Open-source ImmGen: mononuclear phagocytes. Nat Immunol (2016) 17(7):741. doi: 10.1038/ni.3478 27327993

[13] Hoffman D Tevet Y Trzebanski S Rosenberg G Vainman L Solomon A. A non-classical monocyte-derived macrophage subset provides a splenic replication niche for intracellular Salmonella. Immunity (2021) 54(12):2712–23.e6. doi: 10.1016/j.immuni.2021.10.015 34788598 PMC8691386

[14] Monaco G Lee B Xu W Mustafah S Hwang YY Carré C. RNA-seq signatures normalized by mRNA abundance allow absolute deconvolution of human immune cell types. Cell Rep (2019) 26(6):1627–40.e7. doi: 10.1016/j.celrep.2019.01.041 30726743 PMC6367568

[15] Hashimshony T Senderovich N Avital G Klochendler A De Leeuw Y Anavy L. CEL-Seq2: sensitive highly-multiplexed single-cell RNA-Seq. Genome Biol (2016) 17(1). doi: 10.1186/s13059-016-0938-8 PMC4848782 27121950

[16] Kim D Paggi JM Park C Bennett C Salzberg SL. Graph-based genome alignment and genotyping with HISAT2 and HISAT-genotype. Nat Biotechnol (2019) 37(8):907–15. doi: 10.1038/s41587-019-0201-4 PMC7605509 31375807

[17] Dobin A Davis CA Schlesinger F Drenkow J Zaleski C Jha S. STAR: ultrafast universal RNA-seq aligner. Bioinformatics (2013) 29(1):15–21. doi: 10.1093/bioinformatics/bts635 23104886 PMC3530905

[18] Bray NL Pimentel H Melsted P Pachter L. Near-optimal probabilistic RNA-seq quantification. Nat Biotechnol (2016) 34(5):525–7. doi: 10.1038/nbt.3519 27043002

[19] Picard github repository: broad institute of MIT and Harvard (2019). Available at: https://broadinstitute.github.io/picard/.

[20] Beyer M Mallmann MR Xue J Staratschek-Jox A Vorholt D Krebs W. High-resolution transcriptome of human macrophages. PloS One (2012) 7(9):e45466. doi: 10.1371/journal.pone.0045466 23029029 PMC3448669

[21] Love MI Huber W Anders S. Moderated estimation of fold change and dispersion for RNA-seq data with DESeq2. Genome Biol (2014) 15(12). doi: 10.1186/s13059-014-0550-8 PMC4302049 25516281

[22] Robinson MD McCarthy DJ Smyth GK. edgeR: a Bioconductor package for differential expression analysis of digital gene expression data. Bioinformatics (2010) 26(1):139–40. doi: 10.1093/bioinformatics/btp616 PMC2796818 19910308

[23] Ritchie ME Phipson B Wu D Hu Y Law CW Shi W. limma powers differential expression analyses for RNA-sequencing and microarray studies. Nucleic Acids Res (2015) 43(7):e47. doi: 10.1093/nar/gkv007 25605792 PMC4402510

[24] van de Laar L Saelens W De Prijck S Martens L Scott CL Van Isterdael G. Yolk sac macrophages, fetal liver, and adult monocytes can colonize an empty niche and develop into functional tissue-resident macrophages. Immunity (2016) 44(4):755–68. doi: 10.1016/j.immuni.2016.02.017 26992565

[25] Chen H Boutros PC. VennDiagram: a package for the generation of highly-customizable Venn and Euler diagrams in R. BMC Bioinf (2011) 12(1):35. doi: 10.1186/1471-2105-12-35 PMC3041657 21269502

[26] Kolde R. Pheatmap: pretty heatmaps Vol. 1. R package version (2012). p. 726.

[27] Maschalidi S Mehrotra P Keceli BN De Cleene HKL Lecomte K van der Cruyssen R. Targeting SLC7A11 improves efferocytosis by dendritic cells and wound healing in diabetes. Nature (2022) 606(7915):776–84. doi: 10.1038/s41586-022-04754-6 35614212

[28] Zhu T Ge X Gong S Guo S Tao Q Guo J. Prognostic value of lactate transporter SLC16A1 and SLC16A3 as oncoimmunological biomarkers associating tumor metabolism and immune evasion in glioma. Cancer Innovation (2022) 1(3):229–39. doi: 10.1002/cai2.32 PMC10686114 38089757

[29] Tao Q Li X Zhu T Ge X Gong S Guo J. Lactate transporter SLC16A3 (MCT4) as an onco-immunological biomarker associating tumor microenvironment and immune responses in lung cancer. Int J Gen Med (2022) 15:4465–74. doi: 10.2147/IJGM.S353592 PMC9059363 35509603

[30] Hedges JF Kimmel E Snyder DT Jerome M Jutila MA. Solute carrier 11A1 is expressed by innate lymphocytes and augments their activation. J Immunol (2013) 190(8):4263–73. doi: 10.4049/jimmunol.1200732 PMC3622125 23509347

[31] Canonne-Hergaux F Calafat J Richer E Cellier M Grinstein S Borregaard N. Expression and subcellular localization of NRAMP1 in human neutrophil granules. Blood (2002) 100(1):268–75. doi: 10.1182/blood.V100.1.268 12070036

[32] Chen Y-J Lin C-H Ou T-T Wu C-C Tsai W-C Liu H-W. Solute carrier family 11 member A1 gene polymorphisms in reactive arthritis. J Clin Immunol (2007) 27(1):46–52. doi: 10.1007/s10875-006-9050-2 17211726

[33] Singh N Gedda MR Tiwari N Singh SP Bajpai S Singh RK. Solute carrier protein family 11 member 1 (Slc11a1) activation efficiently inhibits Leishmania donovani survival in host macrophages. J Parasitic Diseases (2017) 41(3):671–7. doi: 10.1007/s12639-016-0864-4 PMC5555910 28848257

[34] Lang T Prina E Sibthorpe D Blackwell JM. Nramp1 transfection transfers Ity/Lsh/Bcg-related pleiotropic effects on macrophage activation: influence on antigen processing and presentation. Infect Immun (1997) 65(2):380–6. doi: 10.1128/iai.65.2.380-386.1997 PMC174606 9009286

[35] Stober CB Brode S White JK Popoff JF Blackwell JM. Slc11a1, formerly Nramp1, is expressed in dendritic cells and influences major histocompatibility complex class II expression and antigen-presenting cell function. Infect Immun (2007) 75(10):5059–67. doi: 10.1128/IAI.00153-07 PMC2044529 17620357

[36] Jin J Zhang H Weyand CM Goronzy JJ. Lysosomes in T cell immunity and aging. Front Aging (2021) 2. doi: 10.3389/fragi.2021.809539 PMC9261317 35822050

[37] Cellier M. Developmental control of NRAMP1 (SLC11A1) expression in professional phagocytes. Biology (2017) 6(4):28. doi: 10.3390/biology6020028 28467369 PMC5485475

[38] Ping Y Shan J Liu Y Liu F Wang L Liu Z. Taurine enhances the antitumor efficacy of PD-1 antibody by boosting CD8+ T cell function. Cancer Immunol Immunother (2023) 72(4):1015–27. doi: 10.1007/s00262-022-03308-z PMC10991389 36261540

[39] Meng L Lu C Wu B Lan C Mo L Chen C. Taurine antagonizes macrophages M1 polarization by mitophagy-glycolysis switch blockage via dragging SAM-PP2Ac transmethylation. Front Immunol (2021) 12. doi: 10.3389/fimmu.2021.648913 PMC8071881 33912173

[40] Panda SK Kim D-H Desai P Rodrigues PF Sudan R Gilfillan S. SLC7A8 is a key amino acids supplier for the metabolic programs that sustain homeostasis and activation of type 2 innate lymphoid cells. Proc Natl Acad Sci (2022) 119(46). doi: 10.1073/pnas.2215528119 PMC9674248 36343258

[41] Hodge SH Krauss MZ Kaymak I King JI Howden AJM Panic G. Amino acid availability acts as a metabolic rheostat to determine the magnitude of ILC2 responses. J Exp Med (2023) 220(3). doi: 10.1084/jem.20221073 PMC9794837 36571761

[42] Delaby C Rondeau C Pouzet C Willemetz A Pilard N Desjardins M. Subcellular localization of iron and heme metabolism related proteins at early stages of erythrophagocytosis. PloS One (2012) 7(7):e42199. doi: 10.1371/journal.pone.0042199 22860081 PMC3408460

[43] Qureshi T Sørensen C Berghuis P Jensen V Dobszay MB Farkas T. The glutamine transporter slc38a1 regulates GABAergic neurotransmission and synaptic plasticity. Cereb Cortex (2019) 29(12):5166–79. doi: 10.1093/cercor/bhz055 PMC6918930 31050701

[44] Seegren PV Downs TK Stremska ME Harper LR Cao R Olson RJ. Mitochondrial ca(2+) signaling is an electrometabolic switch to fuel phagosome killing. Cell Rep (2020) 33(8):108411. doi: 10.1016/j.celrep.2020.108411 33238121 PMC7793167

[45] Guo C You Z Shi H Sun Y Du X Palacios G. SLC38A2 and glutamine signaling in cDC1s dictate anti-tumor immunity. Nature (2023) 620:200–8. doi: 10.1038/s41586-023-06299-8 PMC10396969 37407815

[46] Sheng L Luo Q Chen L. Amino acid solute carrier transporters in inflammation and autoimmunity. Drug Metab Dispos (2022) 50(9):1228–37. doi: 10.1124/dmd.121.000705 35152203

[47] Gao H Liang J Duan J Chen L Li H Zhen T. A prognosis marker SLC2A3 correlates with EMT and immune signature in colorectal cancer. Front Oncol (2021) 11. doi: 10.3389/fonc.2021.638099 PMC8240412 34211835

[48] Simpson IA Dwyer D Malide D Moley KH Travis A Vannucci SJ. The facilitative glucose transporter GLUT3: 20 years of distinction. Am J Physiology-Endocrinology Metab (2008) 295(2):E242–E53. doi: 10.1152/ajpendo.90388.2008 PMC2519757 18577699

[49] Chen Y-T Xu X-H Lin L Tian S Wu G-F. Identification of three cuproptosis-specific expressed genes as diagnostic biomarkers and therapeutic targets for atherosclerosis. Int J Med Sci (2023) 20(7):836–48. doi: 10.7150/ijms.83009 PMC10266043 37324184

[50] Jabado N Canonne-Hergaux F Gruenheid S Picard V Gros P. Iron transporter Nramp2/DMT-1 is associated with the membrane of phagosomes in macrophages and Sertoli cells. Blood (2002) 100(7):2617–22. doi: 10.1182/blood-2002-04-1182 12239176

[51] White C Yuan X Schmidt JP Bresciani E Tamika SK Campagna D. HRG1 is essential for heme transport from the phagolysosome of macrophages during erythrophagocytosis. Cell Metab (2013) 17(2):261–70. doi: 10.1016/j.cmet.2013.01.005 PMC3582031 23395172

[52] Alvey CM Spinler KR Irianto J Pfeifer CR Hayes B Xia Y. SIRPA-inhibited, marrow-derived macrophages engorge, accumulate, and differentiate in antibody-targeted regression of solid tumors. Curr Biol (2017) 27(14):2065–77.e6. doi: 10.1016/j.cub.2017.06.005 28669759 PMC5846676

[53] Kopito RR Lodish HF. Primary structure and transmembrane orientation of the murine anion exchange protein. Nature (1985) 316(6025):234–8. doi: 10.1038/316234a0 2410791

[54] Klei TR Meinderts SM van den Berg TK van Bruggen R. From the cradle to the grave: the role of macrophages in erythropoiesis and erythrophagocytosis. Front Immunol (2017) 8:73. doi: 10.3389/fimmu.2017.00073 28210260 PMC5288342

[55] Jain V Yang W-H Wu J Roback JD Gregory SG Chi J-T. Single cell RNA-seq analysis of human red cells. Front Physiol (2022) 13. doi: 10.3389/fphys.2022.828700 PMC9065680 35514346

[56] Palty R Silverman WF Hershfinkel M Caporale T Sensi SL Parnis J. NCLX is an essential component of mitochondrial Na+/Ca2+ exchange. Proc Natl Acad Sci (2010) 107(1):436–41. doi: 10.1073/pnas.0908099107 PMC2806722 20018762

[57] Verma M Wills Z Chu CT. Excitatory dendritic mitochondrial calcium toxicity: implications for Parkinson's and other neurodegenerative diseases. Front Neurosci (2018) 12:523. doi: 10.3389/fnins.2018.00523 30116173 PMC6083050

[58] Jadiya P Cohen HM Kolmetzky DW Kadam AA Tomar D Elrod JW. Neuronal loss of NCLX-dependent mitochondrial calcium efflux mediates age-associated cognitive decline. iScience (2023) 26(3):106296. doi: 10.1016/j.isci.2023.106296 36936788 PMC10014305

[59] Gronski MA Kinchen JM Juncadella IJ Franc NC Ravichandran KS. An essential role for calcium flux in phagocytes for apoptotic cell engulfment and the anti-inflammatory response. Cell Death Differentiation (2009) 16(10):1323–31. doi: 10.1038/cdd.2009.55 PMC2856475 19461656

[60] Ohkura N Yoshiba K Yoshiba N Edanami N Ohshima H Takenaka S. SVCT2-GLUT1-mediated ascorbic acid transport pathway in rat dental pulp and its effects during wound healing. Sci Rep (2023) 13(1). doi: 10.1038/s41598-023-28197-9 PMC9870884 36690706

[61] Peiseler M Kubes P. More friend than foe: the emerging role of neutrophils in tissue repair. J Clin Invest (2019) 129(7):2629–39. doi: 10.1172/JCI124616 PMC6597202 31205028

