# Supplementary material for: Defining solute carrier transporter signatures of murine immune cell subsets
Source: Front Immunol. 2023 Nov 24;14:1276196. doi: 10.3389/fimmu.2023.1276196 (PMC10704505; doi:10.3389/fimmu.2023.1276196)
Supplement: Supplementary file 1 [file DataSheet_1.pdf]

Supplementary Figure S1: Introduction to Triwise dot plots and the data sets used in this manuscript.

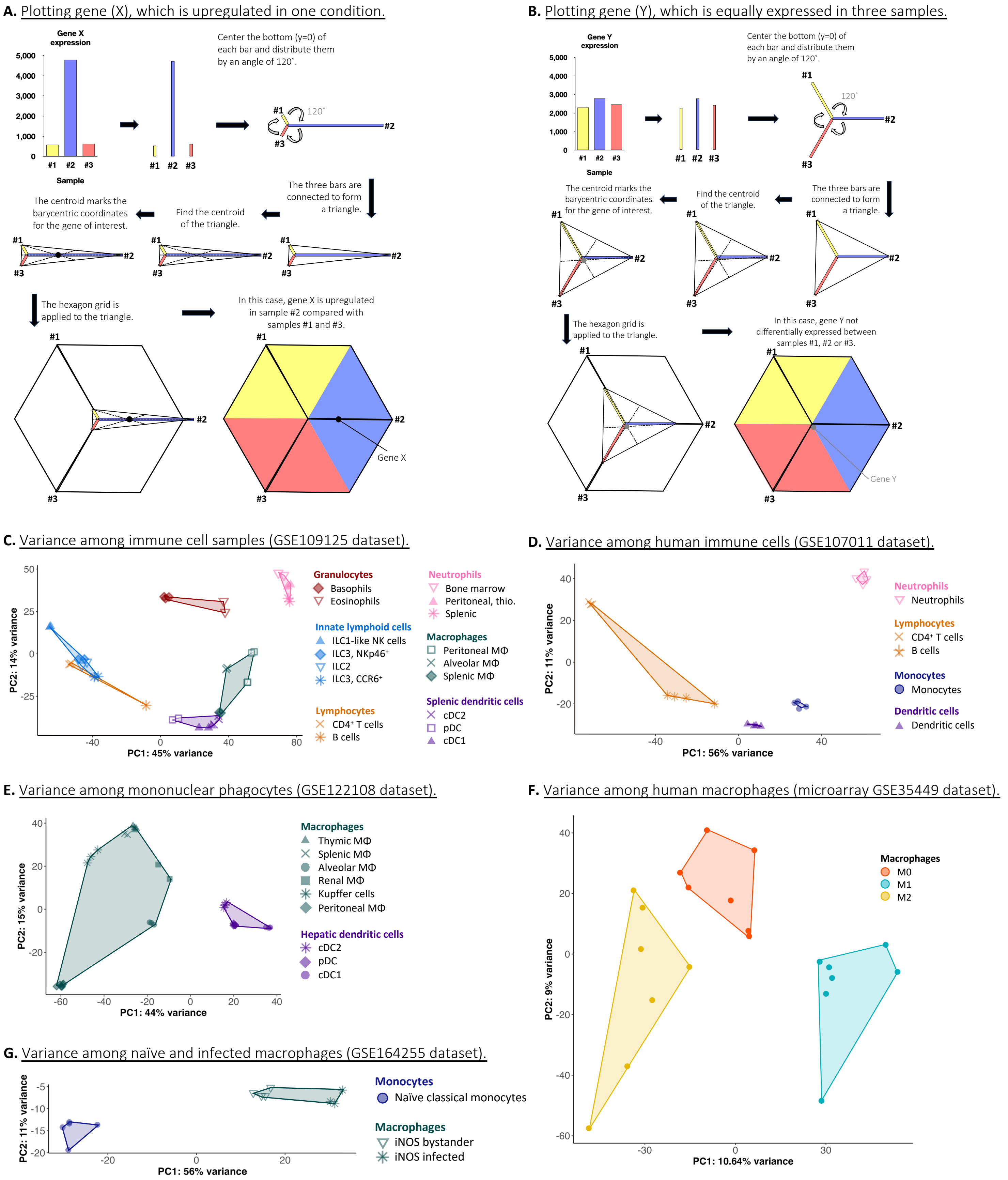

Supplementary Figure S1: Introduction to Triwise dot plots and the data sets used in this manuscript.

(A, B) Two examples that demonstrate how gene expression matrices – here depicted as bar plots – are transformed into barycentric coordinates, which are used the Triwise R package to generate (interactive) dot plots. By centering the bottom of the gene expression bars (y=0) in each sample, and distributing the three bars by angles of 120˚, a triangle can be drawn, by connecting the top (ymax) of the bars. Next, the centroid of the triangle – the intersection between the three medians – is found and marked by a dot, which represents the expression level of one specific gene of interest. By overlaying the triangle on to a hexagon shape, in which each of the three grids represents one sample/cell type, the exact location of the gene and its relative expression level between the three samples is denoted. **(A)** Gene X is an example of a gene, which is higher expressed in just one of the three samples. **(B)** Gene Y represents a gene, which is equally expressed in all three conditions, and which therefore locates to the center of the Triwise dot plot. **(C-G)** PCA plots of the samples analyzed in this manuscript. **(C)** Variance between samples that represent phagocytes, granulocytes, lymphocytes and innate lymphoid cells from the ImmGen GSE109125 data set. **(D)** Variance between RNA-Seq samples of human immune cells – neutrophils, lymphocytes, monocytes and dendritic cells – isolated from PBMCs and deriving from the GSE107011 data set. **(E)** Mononuclear phagocyte samples from the ImmGen GSE122108 data set and their variance represented in a PCA plot. **(F)** Variance between human macrophages at three different differential states: M0, M1 and M2; deriving from the GSE35449 microarray data set. **(G)** Variance among naïve classical monocytes and bystander or *Salmonella*-infected macrophages from the GSE164255 bulk RNA-Seq data set.

Supplementary figure S2: Shared SLC signatures in immune cell populations from the same tissue of origin.

A. Number of overlapping genes among the most expressed SLC transcripts in splenic phagocytes and adaptive immune cells.

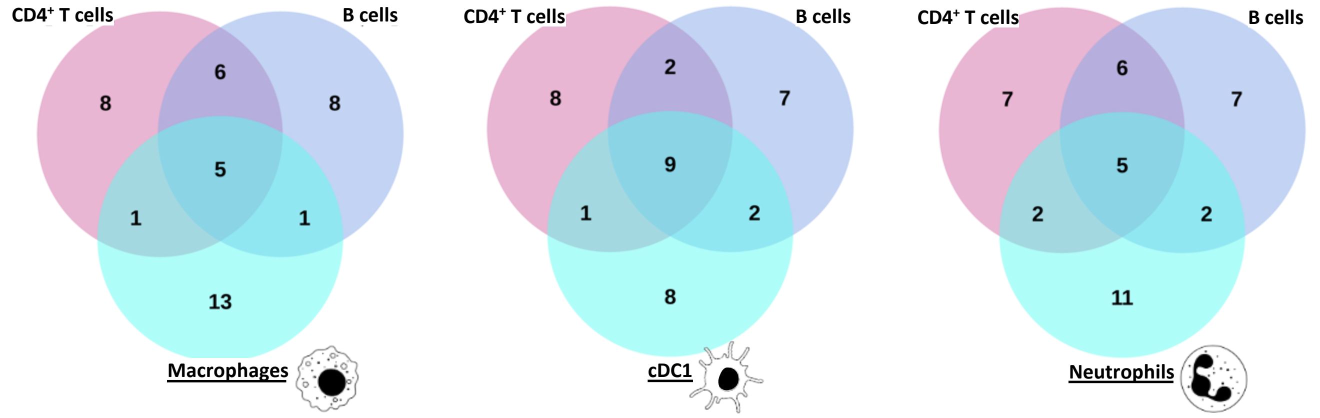

B. Transcript comparison and SLC distribution in innate lymphoid cell (ILC) subtypes across tissues.

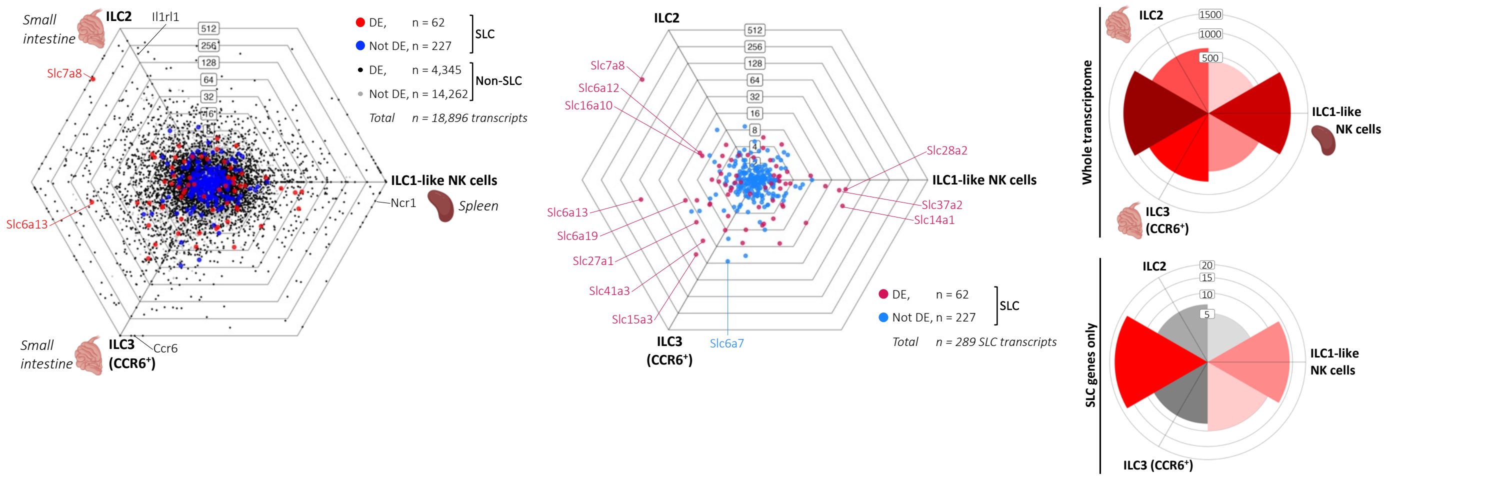

C. Three-way comparison of total transcripts and SLC disctributions of intestinal ILC subtypes -2 and -3.

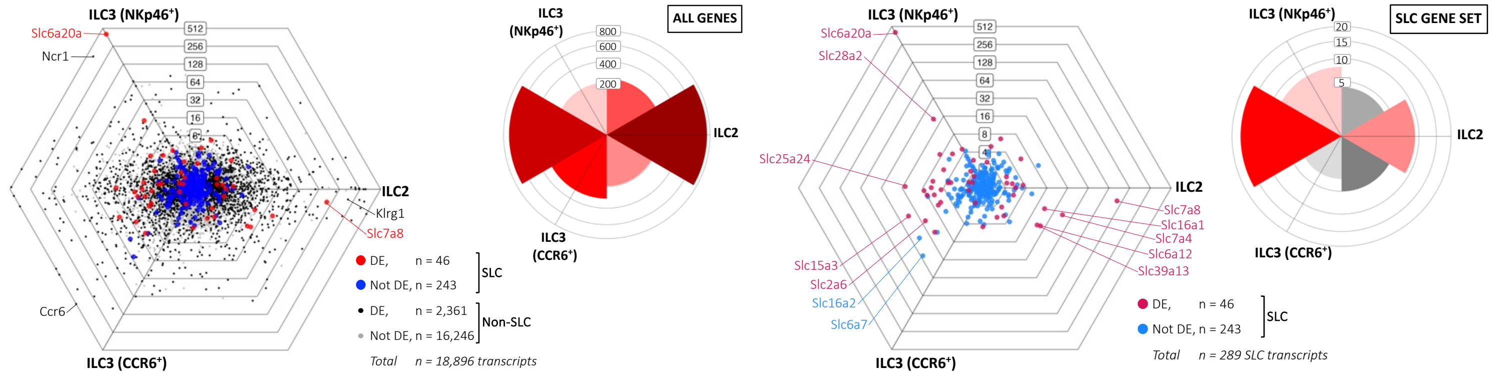

Supplementary figure S2: Shared SLC signatures in immune cell populations from the same tissue of origin.

(A) The top 20 most expressed SLCs of each phagocyte or adaptive immune cell population are compared in a Venn diagram. The lists of unique or overlapping genes can be found in supplementary table S2. **(B)** Triwise dot plot of RNA-Seq data sets representing innate lymphoid cell (ILC) subsets isolated from the spleen or the small intestine (GSE109125). In the left panel, genes are indicated with black dots (•) and SLC-encoding genes with red dots (•) when significantly different expressed. Genes are indicated with grey dots (•) and SLC-encoding genes with blue dots (•) when not differentially expressed. Labels on the dot plot grid lines indicate transcript fold changes (up to 512 folds) of reads in one cellular subset versus another, and genes encoding known markers are indicated: Ilrl1 (ST2), Ncr1 (NKp46) and Ccr6 (C-C Motif Chemokine Receptor 6). In the middle panel, differentially expressed SLCs are depicted in magenta (•) and non-differentially expressed SLCs in light blue (•). Labels on the dot plot grid lines indicate transcript fold changes (up to 512 folds) of reads in one cellular subset versus another, and genes encoding highly expressed SLCs are indicated. In the right panel, rose plots show the directional distribution of all differentially expressed genes (top) or the differentially expressed SLCs specifically (bottom) among the three ILC subsets. The labels on the gridlines indicate the number of genes per rose petal/bucket. **(C)** Triwise dot plot of RNA-Seq data sets representing three ILC subsets isolated from the small intestine (GSE109125). In the left panel, genes are indicated with black dots (•) and SLC-encoding genes with red dots (•) when significantly different expressed. Genes are indicated with grey dots (•) and SLC-encoding genes with blue dots (•) when not differentially expressed. Labels on the dot plot grid lines indicate transcript fold changes (up to 512 folds) of reads in one cellular subset versus another, and genes encoding known markers are indicated: Klrg1 (Killer Cell Lectin Like Receptor G1), Ncr1 (NKp46) and Ccr6 (C-C Motif Chemokine Receptor 6). The associated rose plot shows the directional distribution of all differentially expressed genes among the three ILC subsets. In the right panel, differentially expressed SLCs are depicted in magenta (•) and non-differentially expressed SLCs in light blue (•). Labels on the dot plot grid lines indicate transcript fold changes (up to 512 folds) of reads in one cellular subset versus another, and genes encoding highly expressed SLCs are indicated. The associated rose plot shows the directional distribution of all differentially expressed SLCs specifically among the three ILC subsets. The labels on the gridlines indicate the number of genes per rose petal/bucket.

Supplementary figure S3: Macrophages express unique SLCs independent of the tissue of origin.

A. Total transcript level comparison and directional distribution between macrophages from different tissues of origin.

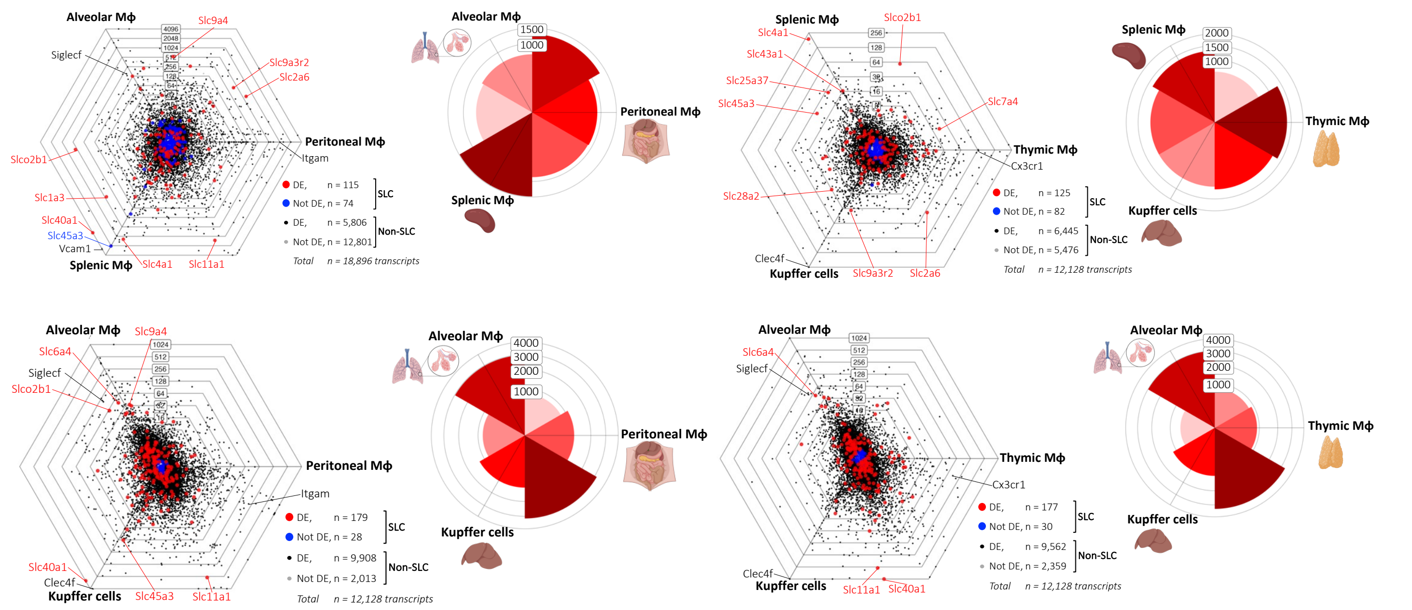

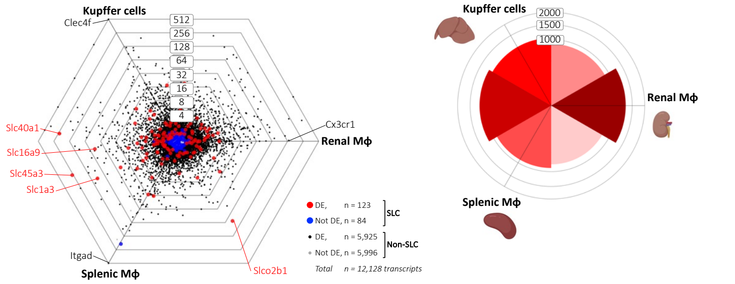

B. SLC transcript clusters in tissue-specific macrophages.

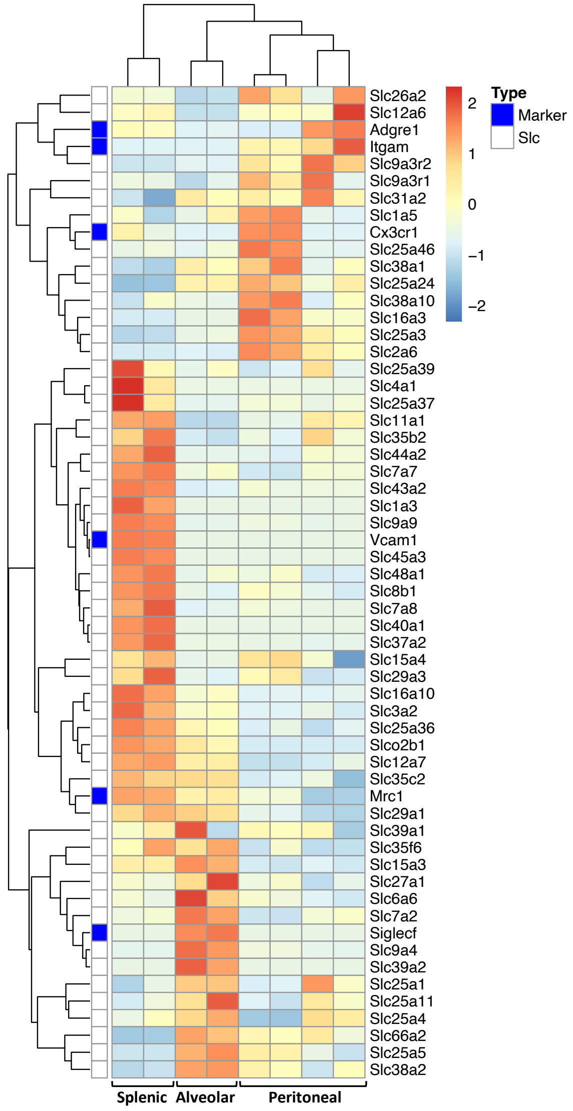

C. Total transcript comparison of M0, M1 and M2 macrophages.

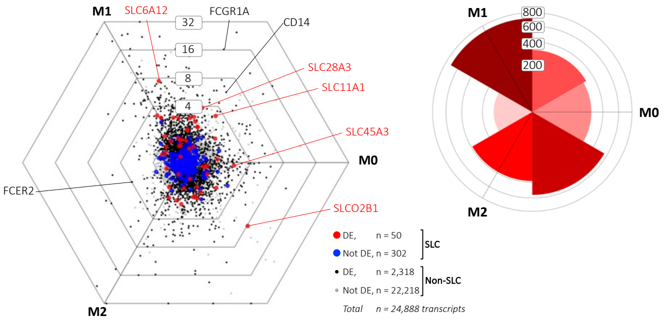

D. Overlapping, highly abundant SLCs in M0, M1 and M2 macrophages.

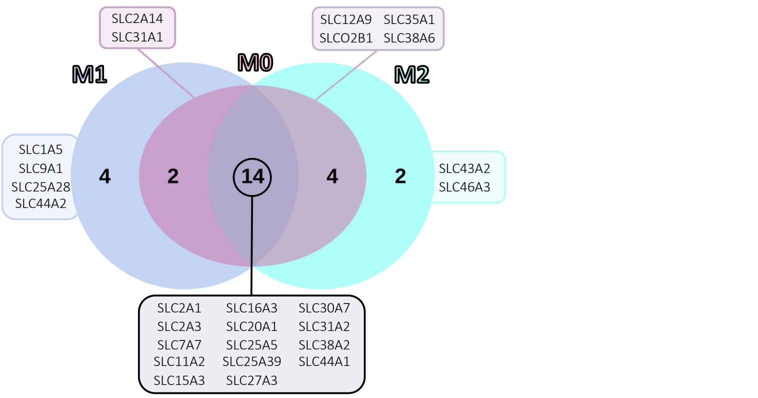

Supplementary figure S3: Macrophages express unique SLCs independent of the tissue of origin.

(A) Triwise dot plots of all transcripts in RNA-Seq data sets from alveolar, peritoneal and splenic macrophages (GSE109125), or from Kupffer cells, splenic, thymic, alveolar, renal or peritoneal macrophages (GSE122108). Known macrophage- or tissue-specific markers are highlighted: Siglecf (Sialic Acid Binding Ig Like Lectin F), Itgam (CD11b), Vcam1 (Vascular Cell Adhesion Molecule 1), Cx3cr1 (C-X3-C Motif Chemokine Receptor 1), Itgad (Integrin Alpha D or Cd11d) and Clec4f (C-Type Lectin Domain Family 4 Member F). **(B)** Normalized reads of the top 20 most expressed SLCs in RNA-Seq data sets (GSE109125) from three macrophage subsets were compared in a heat-map. Clusters of highly expressed SLCs representative of each macrophage subtype are indicated on the y axis, while clusters of tissue origin are indicated on the x axis. The color grading in the heat map ranging from blue to red indicates the relative gene expression. Genes encoding known macrophage-specific markers are highlighted with dark blue boxes: Adgre1 (F4/80), Itgam (CD11b), Cx3cr1 (C-X3-C Motif Chemokine Receptor 1), Vcam1 (Vascular Cell Adhesion Molecule 1), Mrc1 (Mannose Receptor C-Type 1) and Siglecf (Sialic Acid Binding Ig Like Lectin F). **(C)** A Triwise comparison of the total transcriptomes of human M0, M1 and M2 macrophages from a microarray data set (GSE35449). Highlighted macrophage markers are FCER2 (CD23), FCGR1A (CD64) and CD14. **(D)** Venn diagram depicting common and unique SLCs among the top 20 most abundant SLC transcripts in each macrophage subtype. **(A and C)** Genes are indicated with black dots (•) and SLC-encoding genes with red dots (•) when significantly different expressed. Genes are indicated with grey dots (•) and SLC-encoding genes with blue dots (•) when not differentially expressed. Labels on the dot plot grid lines indicate transcript fold changes (up to 4,096 folds) of reads in one cellular subset versus another. The rose plots associated with each respective dot plot indicate the directional distribution of all differentially expressed genes in the Triwise comparison. Gridline labels indicate the number of genes per rose petal/bucket.

Supplementary figure S4: Identifying highly expressed SLCs as potential new markers of dendritic cells subsets.

A. Total transcript level comparison and directional distribution between dendritic cell subsets isolated from spleen and liver.

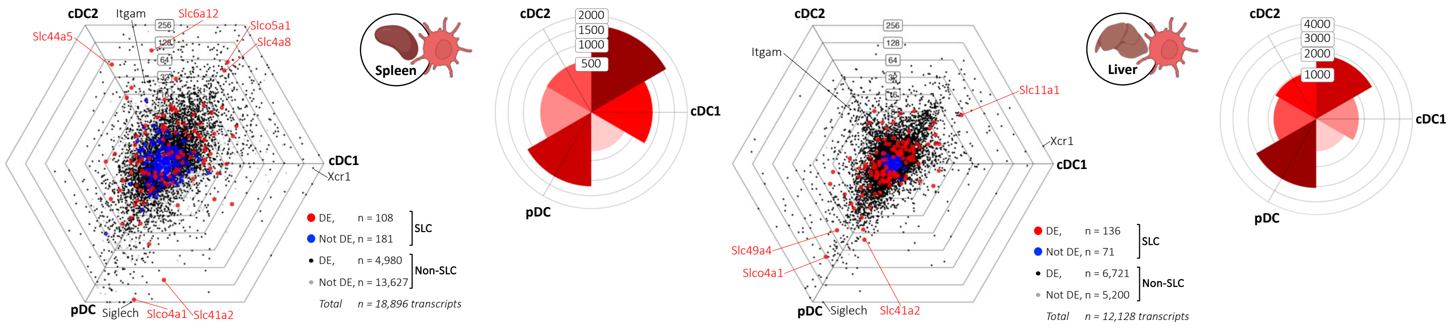

B. Gene expression level of specific, highly abundant SLCs vs known DC markers of dendritic cell subsets.

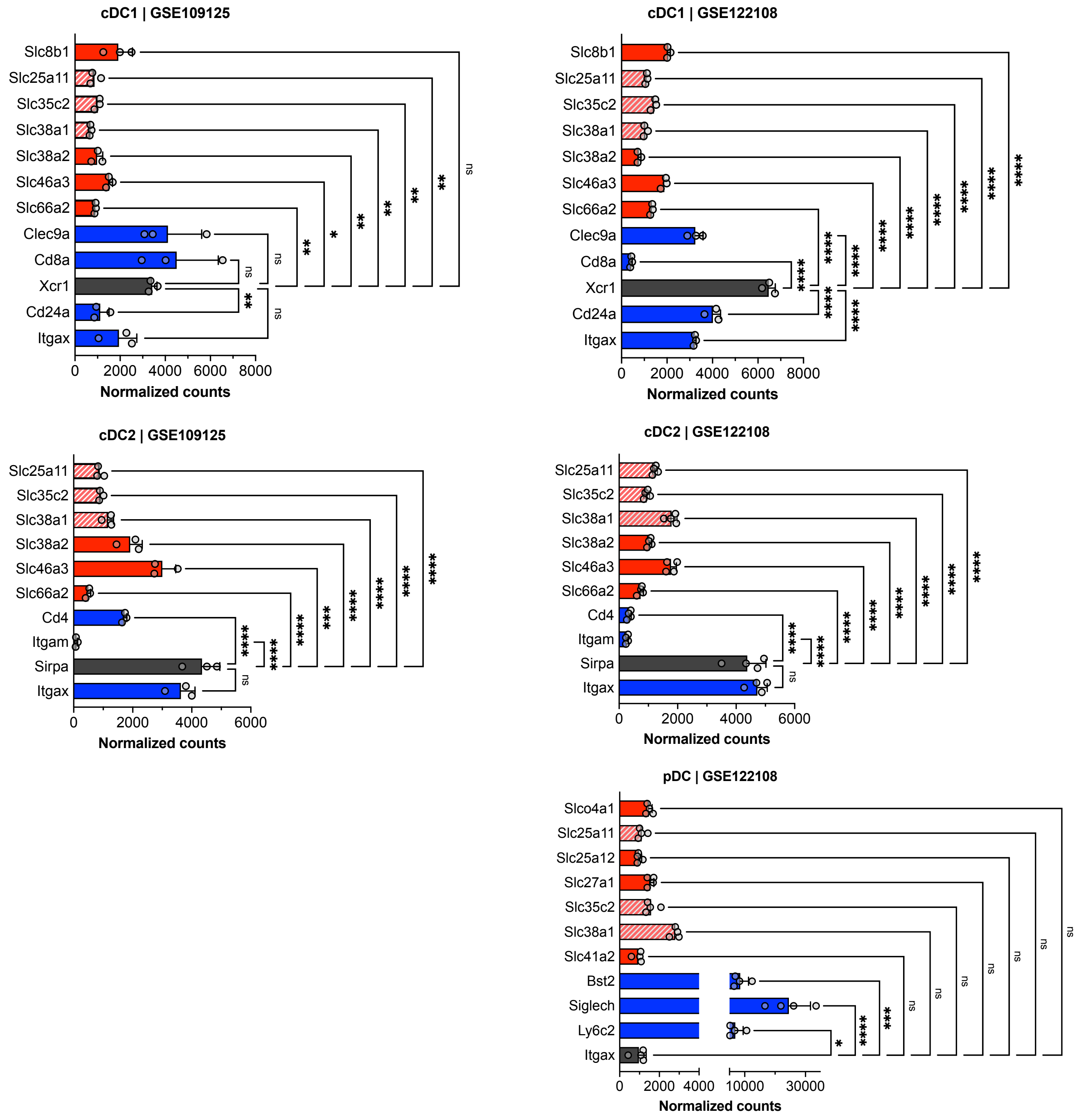

Supplementary figure S4: Identifying highly expressed SLCs as potential new markers of dendritic cells subsets.

(A) Triwise dot plots show the comparison of all transcripts in RNA-Seq data sets from three dendritic cell subsets: cDC1, cDC2 and pDC, which were isolated from either the spleen (GSE109125, left panel) or the liver (GSE122108, right panel). Genes are indicated with black dots (•) and SLC-encoding genes with red dots (•) when significantly different expressed. Genes are indicated with grey dots (•) and SLC-encoding genes with blue dots (•) when not differentially expressed. Labels on the dot plot grid lines indicate transcript fold changes (up to 256 folds) of reads in one cellular subset versus another. Highly and differentially expressed SLCs are labelled along with known DC subtype-specific markers: Itgam (CD11b), Xcr1 (encoding XC motif chemokine Receptor 1) and Siglech (Sialic acid binding Ig-like lectin H). The associated rose plots indicate the directional distribution of all differentially expressed genes in the respective Triwise comparison, spleen or liver. Gridline labels indicate the number of genes per rose petal/bucket. **(B)** Pairwise comparisons between known DC markers versus candidate SLC expression markers expressed as normalized read counts from RNA-Seq data sets (GSE109125 & GSE122108). Each dot represents one biological replicate (= DCs isolated from one mouse) and are plotted as the mean with standard deviation as error bars. Red bars indicate SLCs that are candidate expression markers for that particular DC subtype (cDC1, cDC1 & cDC2, or pDC). Red-white striped bars indicate SLCs, which are highly expressed in all three DC subtypes regardless of their tissue of origin (spleen or liver). Blue and grey bars indicate known DC surface markers (not belonging to the SLC superfamily). The genes represented by the grey bars serve as the control sample for the statistical comparison in each data set. Clec9a (C-type Lectin domain Containing 9A), Cd8a (CD8 subunit Alpha), Xcr1 (XC motif chemokine Receptor 1), Cd24a (HSA), Itgax (CD11c), Cd4 (CD4), Itgam (CD11b), Sirpa (CD172a), Bst2 (Tetherin) and Ly6c2 (Lymphocyte antigen 6 family member C2). Statistics: One-way ANOVA with Dunnett's multiple comparisons test. *, $P < 0.0332$; **, $P < 0.0021$; ***, $P < 0.0002$; ****, $P < 0.0001$; ns = not significant.

Supplementary figure S5: SLC signatures in inflammatory neutrophils and *Salmonella*-infected macrophages.

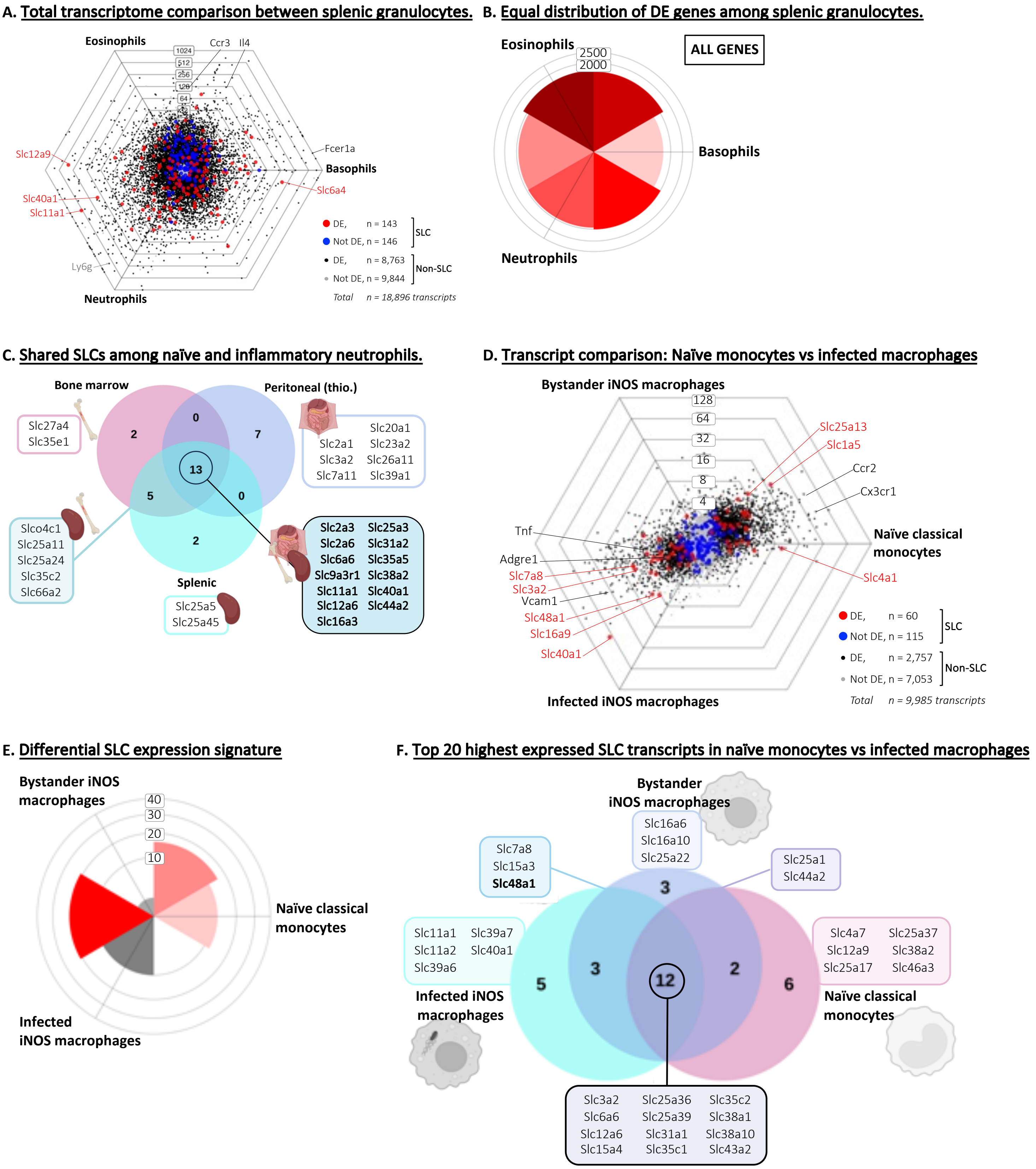

Supplementary figure S5: SLC signatures in inflammatory neutrophils and *Salmonella*-infected macrophages.

(A) Triwise dot plot shows the comparison of all transcripts in RNA-Seq data sets from three splenic granulocyte subsets: Eosinophils, basophils and neutrophils (GSE109125). Highly and differentially expressed SLCs are labelled along with known granulocyte-specific markers: Ccr3 (C-C Motif Chemokine Receptor 3), Il4 (Interleukin 4), Fcer1a (Fc Epsilon (IgE) Receptor Ia) and Ly6g (Ly-6G). **(B)** Rose plot shows the directional distribution of all differentially expressed genes in the Triwise comparison of the three types of granulocytes. Gridline labels indicate the number of genes per rose petal/bucket. **(C)** The 20 most expressed SLCs found in each neutrophil subset – isolated at steady state (bone marrow and spleen) or during inflammation (peritoneal) – are compared in a Venn diagram. The gene names of the SLCs that are unique to one subset or shared among two or all three granulocyte populations are listed in the associated boxes. The 13 SLCs written in **bold font** are among the highest expressed SLCs in all three data sets – regardless of the inflammatory state of the neutrophils. **(D)** Triwise dot plot shows the comparison of all transcripts in RNA-Seq data sets from three splenic subsets: Naïve classical monocytes, bystander iNOS macrophages and infected macrophages from *Salmonella*-infected mice (GSE164255). Highly and differentially expressed SLCs are labelled along with known monocyte- and macrophage-markers: Ccr2 (C-C Motif Chemokine Receptor 2), Cx3cr1 (C-X3-C motif chemokine receptor 1), Tnf (Tumor necrosis factor), Adgre1 (F4/80) and Vcam1 (Vascular Cell Adhesion Molecule 1, Cd106). **(A and D)** Genes are indicated with black dots (•) and SLC-encoding genes with red dots (•) when significantly different expressed. Genes are indicated with grey dots (•) and SLC-encoding genes with blue dots (•) when not differentially expressed. Labels on the dot plot grid lines indicate transcript fold changes (up to 1,024 folds) of reads in one cellular subset versus another. **(E)** Rose plot shows the directional distribution of all differentially expressed SLCs in the Triwise comparison of naïve monocytes vs bystander and infected iNOS macrophages. Gridline labels indicate the number of genes per rose petal/bucket. **(F)** The 20 highest expressed SLCs in each splenic monocyte or macrophage subset are compared in a Venn diagram. The gene names of the SLCs that are unique to one subset or shared among two or all three populations are listed in the associated boxes.
