# Supplementary material for: Defining solute carrier transporter signatures of murine immune cell subsets
Source: Front Immunol. 2023 Nov 24;14:1276196. doi: 10.3389/fimmu.2023.1276196 (PMC10704505; doi:10.3389/fimmu.2023.1276196)
Supplement: Supplementary file 2 [file DataSheet_2.zip › IntDP_Weblinks/intDP3_Aaes et al., Transcript comparison between Neutrophils and lymphocytes.html]

Aaes et al., Transcript comparison between Neutrophils and
lymphocytes
